# Sponging of five tumour suppressor miRNAs by lncRNA-KCNQ1OT1 activates BMPR1A/BMPR1B-ACVR2A/ACVR2B signalling and promotes chemoresistance in hepatocellular carcinoma

**DOI:** 10.1038/s41420-024-02016-0

**Published:** 2024-06-08

**Authors:** Swagata Majumdar, Anannya Chakraborty, Sumit Das, Mahadeo Gorain, Soumyabrata Chatterjee, Indrashish Dey, Sayantani Bhowmik, Suchandrima Ghosh, Sanjana Banerjee, Sk. Mahiuddin Ahammed, Abhijit Chowdhury, Simanti Datta, Gopal Kundu, Soma Banerjee

**Affiliations:** 1 Centre for Liver Research, School of Digestive and Liver Diseases, Institute of Post Graduate Medical Education and Research, Kolkata, West Bengal India; 2 National Centre for Cell Science, Pune, India; 3 Department of Hepatology, School of Digestive and Liver Diseases, Institute of Post Graduate Medical Education and Research, Kolkata, West Bengal India

**Keywords:** Cancer, Cancer stem cells

## Abstract

Diverse mechanisms have been established to understand the chemoresistance of hepatocellular carcinoma (HCC), but the contribution of non-coding RNAs is not surveyed well. Here, we aimed to explore the lncRNA-miRNA axis in Hepatitis C and B virus (HCV and HBV) infected HCC to investigate the molecular mechanism of chemoresistance and to identify a potential therapeutic target for HCC. The small RNA transcriptome analysis followed by qRT-PCR validation with the liver tissues of both HCV and HBV infected HCC patients revealed that miR-424-5p, miR-136-3p, miR-139-5p, miR-223-3p, and miR-375-3p were the most downregulated miRNAs in HCC compared to normal (log_2_ fold change ≤−1.5, *P*_adj_ ≤ 0.05). In silico pathway analysis with the validated targets of each miRNA revealed that the signalling pathway regulating pluripotency of stem cells is commonly targeted by these five miRNAs. Subsequent validation by 3′UTR-luciferase assay and western blot analysis unveiled that these five miRNAs impeded either same or diverse genes, but all linked to BMP signalling pathway such as BMPR1A/BMPR1B by miR-139-5p, miR-136-3p, and miR-375-3p, and ACVR2A/ACVR2B by miR-424-5p and miR-223-3p. Furthermore, restoration of each miRNA in Huh7/SNU449 cells inhibited phosphorylation of downstream SMAD1/5 and ERK1/2, and attenuated Epithelial-mesenchymal transition, stemness, spheroid formation, chemoresistance, invasion and migration of cells. To investigate the mechanism of suppression of these miRNAs, “DIANA” tool was employed and lncRNA-KCNQ1OT1 was retrieved as interacting partner of all the five miRNAs. In vitro RNA pull-down assay revealed that lncRNA-KCNQ1OT1 physically interacted and sequestered these five miRNAs in the cytoplasm. Hence, KCNQ1OT1 was suppressed in Huh7/SNU449 cells using CRISPR technology and observed regression of oncogenic properties with enhanced chemosensitivity and reduced metastasis in cancer cells. Shrinkage of tumour size and volume in NOD-SCID mice injected with KCNQ1OT1-sgRNA cells further strengthened our observations. Thus, lncRNA-KCNQ1OT1 is the main regulator, which reduces the level of beneficiary miRNAs in the tumour milieu and modulates BMP signalling pathway to promote chemoresistance in HCC, suggesting lncRNA-KCNQ1OT1 might have robust potential to be a therapeutic target in HCC.

## Introduction

Hepatocellular carcinoma (HCC) is one of the most aggressive primary liver cancers accounting for the 3^rd^ highest cancer-related mortality worldwide [1, 2]. Decades of chronic infection with hepatitis B and hepatitis C virus (HBV and HCV) are the major risk factors for HCC [3]. Unfortunately, curative therapy can only be given to a minority of HCC patients. The complex molecular heterogeneity is the major hurdle in offering therapy to advanced HCC patients who are often chemo-resistant [4, 5]. Sorafenib is the only approved drug that could extend life expectancy of these patients for 4–6 months [6]. This evokes the necessity of identification of new promising therapeutics and more research to understand the pathophysiological mechanisms of HCC in order to develop improved therapeutic strategy. The cancer stem cells (CSC) like features of cancer cells are responsible for such chemoresistance [7]. Hence, molecular level understanding of pathways driving CSC-like features is required to identify effective therapeutics for HCC. In various cancers, several studies have highlighted the importance of bone morphogenetic proteins (BMPs) in modulating proliferation, invasion and metastasis during carcinogenesis [8]. In this context, the secreted cytokines BMP4 and BMP2 have been shown to function through type I receptors BMPR1A/BMPR1B and type II receptors ACVR2A/ACVR2B [9] and Chiu CY et al. have established that BMP4 and BMPR1A type I receptor promote proliferation and metastasis in HCC through SMAD1/5/8 independent ERK1/2 pathway [10]. A few studies have showed that BMP4 and its receptor BMPR1A are overexpressed in various cancers and promote CSC-like features [11, 12], but the detailed regulatory mechanism is not clearly known.

Non-coding RNAs (ncRNAs), including microRNAs (miRNAs, 18–22nt long) and long non-coding RNAs (lncRNAs, >200nt long), have gained attention as major epigenetic regulators of almost all cellular pathways [13]. Several groups have constructed disease-specific differentially expressed miRNA-lncRNA-mRNA regulatory network [14], but BMP signalling pathway remains less explored. In 2015, Li L et al. have established that the aggressiveness of HCC is associated with active hsa-miR-148a-ACVR1-BMP-WNT circuit [15].

In this context, we studied for the first time, the detailed molecular mechanism of the mRNAs-miRNAs-lncRNA network in BMP signalling pathway which is required for the maintenance of CSC-like features in HCC cells. Five most downregulated tumour suppressor miRNAs (miR-424-5p, miR-136-3p, miR-139-5p, miR-223-3p, and miR-375-3p) were retrieved from the small RNA transcriptome data of liver tissue samples from HCC patients. Target recognition and pathway analyses revealed that these five miRNAs commonly impeded the pluripotency of stem cell signalling pathways where their targets were BMP receptors only, such as BMPR1A/1B and ACVR2A/2B. Restoration of each miRNA inhibited both SMAD1/5 and ERK1/2 pathways in Huh7 and SNU449 cells and prevented Epithelial to Mesenchymal transition (EMT), stemness, spheroid formation, invasion, migration, proliferation, and chemoresistance. Furthermore, these miRNAs were found to be sequestered by a single lncRNA, KCNQ1OT1 in the cytoplasm of HCC cells. Hence, suppression of KCNQ1OT1 using CRISPR tool [16] in Huh7 and SNU449 cells restored functions of all five miRNAs and induced chemosensitivity in HCC cells. In addition, shrinkage of tumour size was noted when these cells were implanted to the flanks of NOD/SCID mice suggesting KCNQ1OT1 could be a potent therapeutic target in HCC.

## Results

### Profiling of downregulated miRNAs in the liver tissue of the hepatitis virus-infected HCC

**Fig. 1:**
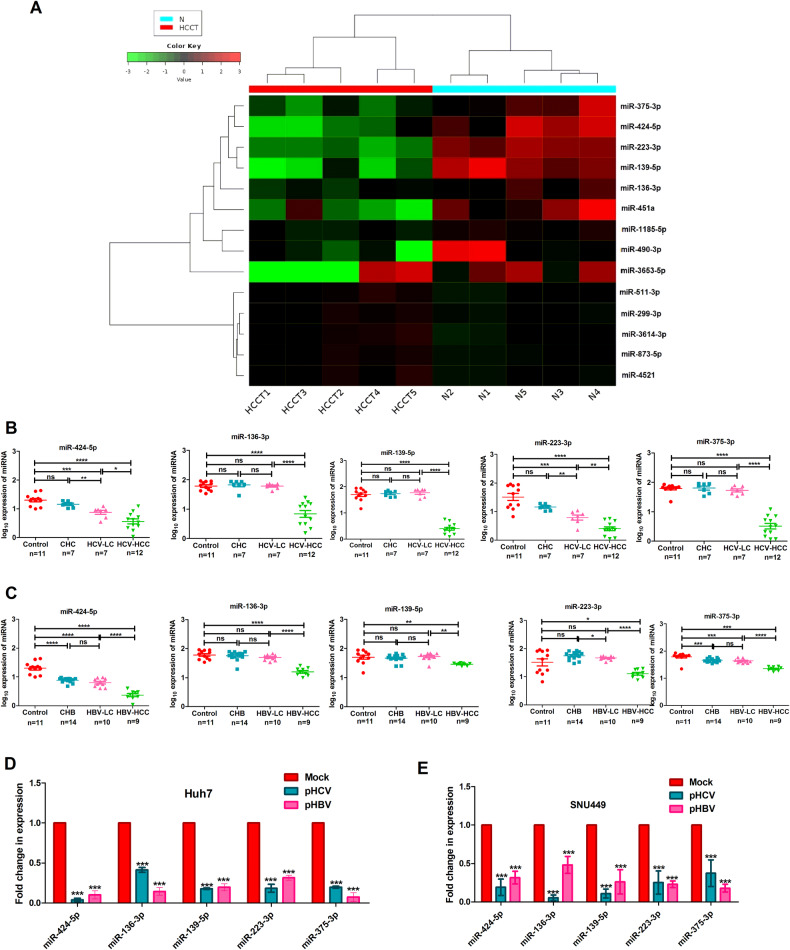
Selection and validation of downregulated miRNAs in HCC liver tissue. (**A**) Heatmap represents the downregulated miRNAs in liver tissues of HCV-HCC (*n* = 5) vs. Control (*n* = 5). qRT-PCR analysis was performed with liver tissue samples from progressive disease stages of (**B**) HCV infection and (**C**) HBV infection including chronic hepatitis C (CHC) or chronic hepatitis B (CHB), liver cirrhosis (LC), and hepatocellular carcinoma (HCC) and normal (control). In vitro validation of the expression of the five downregulated miRNAs in (**D**) Huh7 and (**E**) SNU449 cells transfected with HCV and HBV producing plasmids, pS52/JFH1 (pHCV) and pSV2neoHBV2x (pHBV) vs. mock. *p* value was calculated using Mann–Whitney test for **B**, **C** and unpaired student’s *t*-test for **D**, **E**. *, **, ***, and **** indicate *p* value < 0.05, <0.01, <0.001, and <0.0001, respectively. ns indicates not significant.

The small RNA transcriptome profiling of liver tissue samples from HCV-infected HCC patients (*n* = 5) compared to control individual (*n* = 5) revealed that miR-424-5p, miR-136-3p, miR-139-5p, miR-223-3p, and miR-375-3p were the most downregulated miRNAs in HCC (log_2_fold change ≤−1.5, *P*_adj_ ≤ 0.05) as presented in the heatmap (Fig. 1A). The data was also verified using GEO datasets (GSE21362, GSE40744, and GSE74618) with liver tissue of HCC and adjacent normal (Table S6). To understand the molecular mechanism of repression of these tumour suppressor miRNAs in HCC, their expression patterns were assessed at different progressive stages of liver diseases from chronic hepatitis C (CHC) to HCC through liver cirrhosis (LC) by qRT-PCR. The results clearly demonstrated that the expression of these miRNAs remained unaltered between normal and CHC. However, their expressions were significantly reduced at the end-stage liver diseases such as LC (miR-424-5p/miR-223-3p) and HCC (miR-424-5p/miR-136-3p/miR-139-5p/miR-223-3p/miR-375-3p) (Fig. 1B).

Consistent with this data, a similar diminishing trend in the expression of these miRNAs was also observed at different progressive stages of HBV-infected samples such as chronic hepatitis B (CHB) (miR-424-5p/miR-375-3p), HBV-LC (miR-424-5p/miR-136-3p/miR-375-3p), and HBV-HCC (miR-424-5p/miR-136-3p/miR-139-5p/miR-223-3p/miR-375-3p) compared to normal (Fig. 1C). The data was validated after transfection of pS52/JFH1 (pHCV) and pSV2neoHBV2x (pHBV) in Huh7 and SNU449 cells (Fig. 1D, E).

### Identification and validation of pathways targeted by the five downregulated miRNAs in HCC

**Fig. 2:**
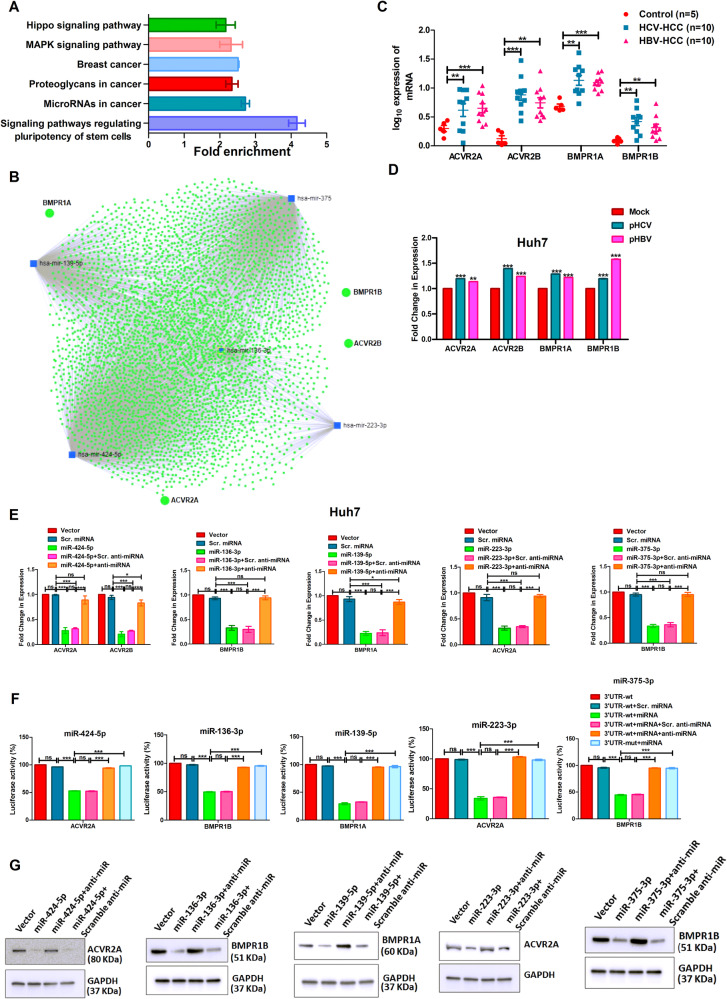
Identification of pathway(s) commonly targeted by downregulated miRNAs and validation of targets. (**A**) Graphical representation of KEGG pathway analysis with validated target genes of five miRNAs. (**B**) Integrated analysis of the target genes of the five miRNAs using miRNet. Expression analysis of target genes by qRT-PCR using (**C**) liver tissues of HCV and HBV infected HCC patients vs. control, and (**D**) Huh7 cells transfected with HCV (pS52/JFH1) and HBV (pSV2neoHBV2x) producing plasmids for 48 h. (**E**) The expression of target genes were determined in Huh7 cells transfected with pRNAU6.1 (vector), pScramble Pre-miRNA (Scr. miRNA), pPre-miRNA (miRNA), pPre-miRNA + scramble anti-miRNA oligo (Scr. anti-miRNA), or pPre-miRNA + anti-miRNA oligo (anti-miRNA) independently. (**F**) 3′UTR-Luciferase assay using Huh7 cells transfected with either wild-type-3′UTR-Luciferase construct (3’UTR-wt) alone or with pScramble pre-miRNA, pPre-miRNA, pPre-miRNA + scramble anti-miRNA oligo, pPre-miRNA + anti-miRNA oligo, separately and mutated-3′UTR-luciferase construct (3′UTR-mut) + pPre-miRNA. (**G**) Immunoblot analysis with anti-ACVR2A, anti-BMPR1B, anti-BMPR1A, and anti-GAPDH-HRP using lysates of Huh7 cells transfected with pRNAU6.1, pPre-miRNA, pPre-miRNA + anti-miRNA oligo, and pPre-miRNA + scramble anti-miRNA oligo. *p* value was calculated using Mann–Whitney test for **C** and unpaired student’s *t*-test for **D**–**F**. *, **, *** indicate *p*-value < 0.05, <0.01, and <0.001 respectively. ns means not significant.

The potential targets of the five downregulated miRNAs were identified by in silico analysis using three independent bioinformatics tools such as TargetScan, miRDB, and micro-T-CDS, and the expression of each of the target gene was verified from TCGA–LIHC datasets to select the putatively overexpressed target genes in HCV and HBV infected HCC (Table S7). The selected target genes were then subjected to the KEGG pathway analysis and observed that the pathways regulating pluripotency of stem cell signalling was the most enriched pathway (FDR ≥ 3 and *p* value ≤ 0.05) commonly targeted by these five miRNAs independently (Fig. 2A and S1A–E). The target genes were ACVR2A (by miR-424-5p and miR-223-3p), ACVR2B (by miR-424-5p), BMPR1A (by miR-139-5p), BMPR1B (by miR-136-3p and miR-375-3p) receptors of BMP signalling pathway [17]. The integrated interactions among these miRNAs and target genes were also verified using miRNet analysis (Fig. 2B).

The impact of BMP signalling pathway in HCC is not well established. To understand the molecular mechanism, the expression of these four genes in HCC liver tissues was further validated by qRT-PCR and observed that BMPR1A and ACVR2B were overexpressed in >80% HCC cases while BMPR1B and ACVR2A were higher in ~60% samples (Fig. 2C). All the four genes were upregulated in Huh7 and SNU449 cells transfected with either pS52/JFH1 (pHCV) or pSV2neoHBV2x (pHBV) (Figs. 2D and S1F). The expression of each target gene was found to be diminished significantly upon overexpression of the respective miRNAs by transfecting pPre-miRNA in Huh7 and SNU449 cells independently and compared with pScramble pre-miRNA transfected cells. It restored back to the control level upon addition of each miRNA-specific anti-miRNA oligo while the scramble anti-miRNA oligo showed no effect on suppression of miRNA activity. There was no significant alteration in gene expression between control-vector (pRNAU6.1) and pScramble pre-miRNA transfected cells (Figs. 2E and S1G).

### 3′UTR-Luciferase assays to confirm binding of miRNAs to the corresponding target gene

Huh7 cells were transfected with either 3′-UTR-Luciferase-reporter construct alone or with pScramble pre-miRNA, pPre-miRNA, pPre-miRNA + scramble anti-miRNA oligo, pPre-miRNA + miRNA-specific anti-miRNA oligo. The luciferase activity was reduced more than 50% in each case of pPre-miRNA transfected cells compared to scramble pre-miRNA independently and the reduced luciferase activity was maintained in presence of pPre-miRNA with scramble anti-miRNA oligo but it restored back to control level upon miRNA-specific anti-miRNA oligo treatment (Fig. 2F). There was no suppression of luciferase activity when a luciferase construct with mutated miRNA-binding site was transfected with pPre-miRNA.

The inhibitory effect of each miRNA to their corresponding target gene was also verified at the protein level by immunoblot analysis with respective antibody and observed that overexpression of miRNA suppressed its target protein expression which was restored back upon miRNA-specific anti-miRNA oligo treatment. The scramble anti-miRNA oligo showed no effect on miRNA activity. (Fig. 2G).

### Effect of miRNAs on BMP signalling which induces CSC-like features in HCC cells

**Fig. 3:**
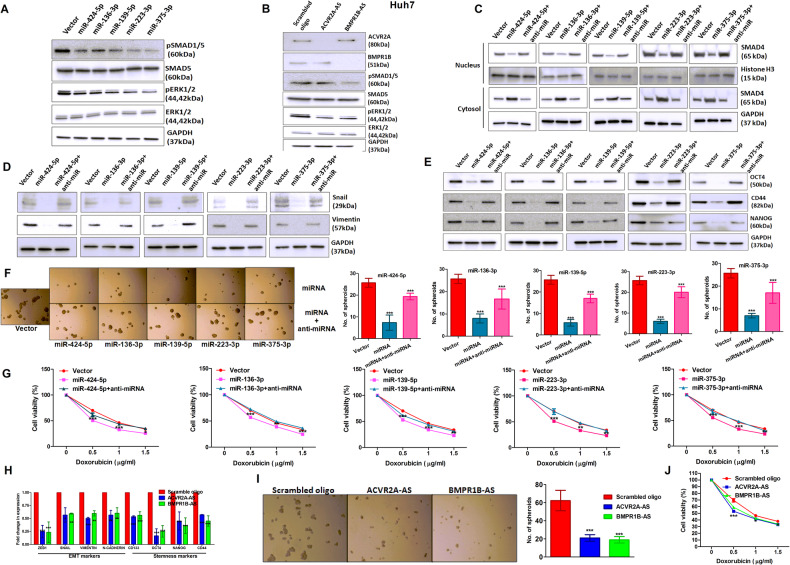
Impact of five miRNAs on BMP signalling cascade. Immunoblot analysis with cell lysates of Huh7 cells transfected with **(A**) vector (pRNAU6.1) and five miRNAs independently, and (**B**) scrambled oligo, ACVR2A-AS, and BMPR1B-AS oligo to verify the expression of total SMAD5, p-SMAD5, ERK1/2, and p-ERK1/2. GAPDH was an internal loading control. (**C**) The expression analysis of SMAD4 in the purified nuclear and cytoplasmic fraction of Huh7 cells transfected with vector, pPre-miRNA, and pPre-miRNA + anti-miRNA oligo. Histone H3 and GAPDH were used as loading control for nuclear and cytoplasmic fraction respectively. Huh7 cells were transfected with vector, pPre-miRNA, and pPre-miRNA + anti-miRNA oligo, and assessed the expression of (**D**) EMT markers (SNAIL and Vimentin) and (**E**) Stemness markers (OCT4, CD44, and NANOG) by immunoblot analysis, (**F**) quantified spheroid number on ultralow attachment plate, and (**G**) detected doxorubicin sensitivity. Huh7 cells were transfected with vector, ACVR2A-AS, and BMPR1B-AS and quantified (**H**) the expression of EMT markers (ZEB1, SNAIL, VIMENTIN, E-CADHERIN) and Stemness markers (CD133, OCT4, NANOG, CD44) by qRT-PCR, (**I**) spheroid number, and (**J**) doxorubicin sensitivity. *p* value was calculated using unpaired student’s *t*-test for **F**, **G**, **I**, **J**, and two-way ANOVA for **H**. *, **, *** indicate *p* value < 0.05, <0.01, and <0.001, respectively.

To evaluate the impact of each miRNA on BMP signalling axis, Huh7 and SNU449 cells were transfected with each pPre-miRNA independently and immunoblot analysis with the cell lysates showed a significant reduction in phosphorylated form of both SMAD1/5 and ERK1/2 in miRNA overexpressing cells compared to empty vector-transfected cells, while the basal levels of SMAD1/5 and ERK1/2 were unaltered (Figs. 3A and S2A). In addition, immunoblot analysis of the purified cytoplasmic and nuclear fraction showed a significant reduction in nuclear SMAD4 level and a comparable increase in its cytoplasmic level was noted upon overexpression of each miRNA. The data was reversed after treatment with miRNA-specific anti-miRNA oligo. (Fig. 3C). In line with miRNA overexpression, reduced expression of phospho-SMAD1/5 and phospho-ERK1/2 was also observed in Huh7 and SNU449 cells transfected with anti-sense (AS) oligo of ACVR2A (ACVR2A-AS) and BMPR1B (BMPR1B-AS) compared to scramble oligo (Figs. 3B and S2B).

Next, the expression of EMT markers including ZEB1, SNAIL, Vimentin, N-Cadherin and stem cell markers such as CD133, CD44, OCT4, NANOG were examined in Huh7 and SNU449 cell lines by qRT-PCR and immunoblot analysis respectively. Each of these markers was reduced upon overexpression of individual miRNA compared to vector and re-established again upon miRNA-specific anti-miRNA oligo treatment (Figs. S2C and 3D, E). Keeping similar setting of transfection, the numbers and sizes of the spheroid were noted to be decreased markedly upon overexpression of individual miRNA in both Huh7 and SNU449 cells grown on ultralow attachment plates compared to vector and miRNA-specific anti-miRNA oligo treatment (Fig. 3F and S2E). The miRNA over-produced Huh7 and SNU449 cells also became more sensitive to doxorubicin than vector control and anti-miRNA oligo treatment (Figs. 3G and S2F). Similar findings were observed upon treatment of Huh7 and SNU449 cells with ACVR2A-AS and BMPR1B-AS independently compared to scramble oligo treated cells. The expression of EMT and stemness markers were dampened (Figs. 3H and S2G), less spheroids were formed (Figs. 3I and S2H) and cells became more sensitive to doxorubicin upon treatment with ACVR2A-AS and BMPR1B-AS compared to scramble oligo (Figs. 3J and S2I). Thus, these data strongly demonstrate the function of each miRNA through BMP signalling axis.

### Impact of miRNAs on cancer cell proliferation, invasion and metastatic potential

**Fig. 4:**
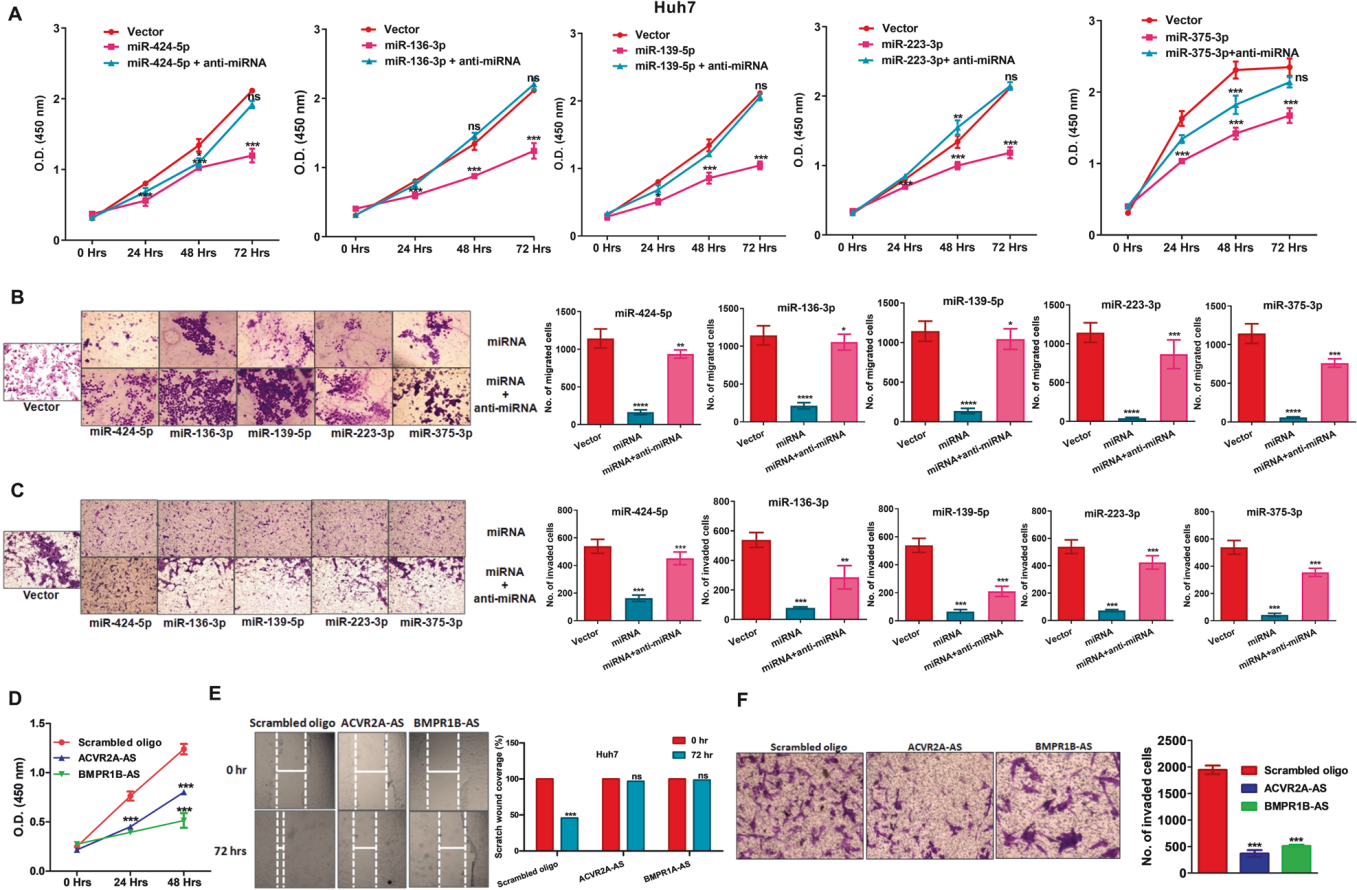
Effect of miRNAs on proliferation, migration and invasion of Huh7 cells. Huh7 cells were transfected with vector, pPre-miRNA, pPre-miRNA + anti-miRNA oligo and determined (**A**) cell proliferation at 0, 24, 48, and 72 h, (**B**) migration of cells in Boyden chamber at 24 h, and (**C**) invasion of cells through Matrigel coated Boyden chamber at 48 h. Huh7 cells were transfected with Scramble oligo, ACVR2A-AS and BMPR1B-AS oligo, and quantified (**D**) cell proliferation, (**E**) cell migration using wound-healing assay, and (**F**) cell invasion in Matrigel coated Boyden chamber. *p* value was calculated using unpaired student’s t-test for **A**–**F**. *, **, ***, **** indicate *p* value < 0.05, <0.01, <0.001, and <0.0001, respectively. ns means not significant.

The pro-metastatic function of BMP signalling has been reported by many groups [15]. Here, we also observed that the proliferation, migration and invasion were reduced drastically upon overexpression of individual miRNA in Huh7 and SNU449 cells, while reverse data was obtained upon addition of miRNA-specific anti-miRNA oligo in WST1 assay, wound-healing assay and Boyden chamber assay in absence or presence of Matrigel respectively (Figs. 4A and S3A–C; Figs. 4B, C and S3D). Treatment of ACVR2A-AS and BMPR1B-AS in both Huh7 and SNU449 cells also showed suppression of proliferation, migration, and invasion compared to scramble oligo treated cells (Figs. 4D and S4A; Figs. 4E and S4B; Figs. 4F and S4C).

Thus, our observations clearly reinforce that BMP signalling is under surveillance of five tumour suppressor miRNAs, downregulation of which triggers CSC-like features in HCC cells.

### LncRNA-KCNQ1OT1 is the common regulator of all five miRNAs

**Fig. 5:**
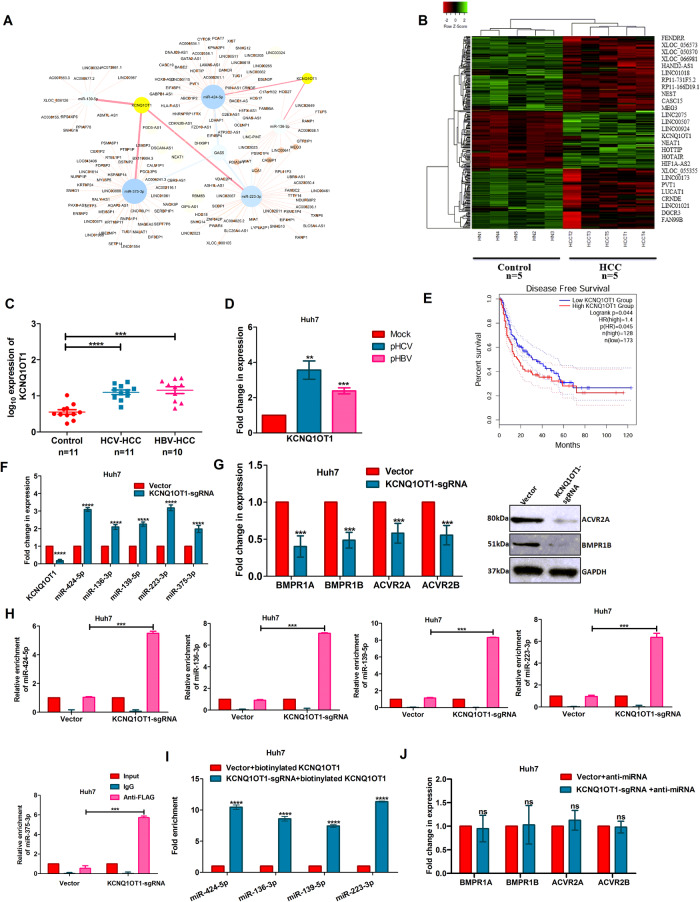
LncRNA-KCNQ1OT1 sponges five downregulated miRNAs. (**A**) Cytoscape analysis was performed to verify interactions among five miRNAs and the predicted long non-coding RNA (lncRNAs). (**B**) A hierarchical clustering analysis with the deregulated lncRNAs in the liver tissue of HCV-HCC (*n* = 5) vs. Control (*n* = 5). Expression analysis of KCNQ1OT1 in (**C**) liver tissues of control, HCV and HBV-infected HCC, and (**D**) HCV and HBV-infected Huh7 cells. (**E**) Overall disease-free survival analysis using TCGA–LIHC data. KCNQ1OT1 was deleted in Huh7 cells using CRISPR tool and quantified the expression of (**F**) five miRNAs and (**G**) their target genes by qRT-PCR and immunoblot analysis. (**H**) RNA-immunoprecipitation (RIP) assay with the cell lysates of pAgo2-FLAG transfected Cas9-Huh7 and KCNQ1OT1-sgRNA-Huh7 cells that were immuno-precipitated with anti-FLAG antibody followed by quantified each miRNA in the pulled down RNA-Protein complex by qRT-PCR. (**I**) In vitro Biotin-Streptavidin pull down assay with Cas9-Huh7 and KCNQ1OT1-sgRNA-Huh7 cell lysates incubated with biotinylated lncRNA clones followed by qRT-PCR analysis of mature miRNAs. (**J**) KCNQ1OT1-sgRNA-Huh7 cells were transfected with pool of five anti-miRNAs and quantified each target gene. *p* value was calculated using unpaired student’s *t*-test for **C**–**J**. **, ***, **** indicate *p* value < 0.01, <0.001, and <0.0001 respectively. ns means not significant.

To investigate the mechanism of suppression of these beneficiary miRNAs during carcinogenesis, both transcription and post-transcriptional regulatory mechanisms were explored. Surprisingly, there was no significant differences in expression at pre-miRNA level for all five miRNAs between HCC and control samples (Fig. S5A). But, LncRNA-miRNA interaction analysis using “lncBase V3.0-DIANA” tool revealed that only lncRNA-KCNQ1OT1 had binding sites for all five miRNAs, and subsequent network analysis using Cytoscape tool supported these interactions (Fig. 5A). This lncRNA was also noted among the most upregulated lncRNAs (log_2_Fc > 2, *p* < 0.05) in mRNA transcriptome data of our HCV-HCC samples (GSE140845) (Fig. 5B). Higher expression of KCNQ1OT1 was validated by qRT-PCR analysis using HCV-HCC and HBV-HCC samples (Fig. 5C) and in Huh7 and SNU449 cell lines transfected with either pS52/JFH1 or pSV2neoHBV2x compared to control (Figs. 5D and S5B). In addition, subcellular fractionation revealed that KCNQ1OT1 was abundantly present in both nuclear and cytoplasmic fraction of both Huh7 and SNU449 cells (Fig. S5C). High level of KCNQ1OT1 observed to be linked with poor disease-free survival in HCC patients (Fig. 5E). The Pearson’s correlation plots also depicted a negative association between expression of each miRNA and KCNQ1OT1 in HCC patients of our cohort (Fig. S5D).

### LncRNA-KCNQ1OT1 sponges miRNAs as an endogenous competitive RNA

To further verify lncRNA-miRNA interaction, KCNQ1OT1 was silenced in both Huh7 and SNU449 cells using CRISPR-Cas9 technology and observed that the expression of each miRNA was recovered while their targets being suppressed in KCNQ1OT1-sgRNA-Huh7/SNU449 cells compared to Cas9-Huh7/SNU449 cells (Figs. 5F and S5E, Figs. 5G and S5F). Next, RNA immuno-precipitation (RIP) analysis was performed with anti-FLAG antibody using pAgo2-FLAG overexpressed Cas9-Huh7/SNU449 and KCNQ1OT1-sgRNA-Huh7/SNU449 cells. Following immunoprecipitation and RNA extraction, qRT-PCR analysis revealed enrichment of each miRNA to the RNA-inducing silencing complex (RISC) in KCNQ1OT1-sgRNA-Huh7/SNU449 cells compared to Cas9-Huh7/SNU449 cells (Figs. 5H and S5G).

The direct interaction between lncRNA and each of the five miRNAs was also verified. Here, lncRNA clones encompassing miRNA-binding sites were in vitro transcribed, biotinylated (both sense and anti-sense) and incubated with the cell lysates of Cas9-Huh7/SNU449 and KCNQ1OT1-sgRNA-Huh7/SNU449. The ribo-complex was precipitated with streptavidin beads, RNA was extracted and quantified. The qRT-PCR data showed enrichment of all miRNAs (miR-424-5p, miR-136-3p, miR-139-5p, and miR-223-3p) in ribo-complex precipitated with sense lncRNA only in KCNQ1OT1-sgRNA-Huh7/SNU449 cells compared to control cells (Figs. 5I and S5H), while anti-sense lncRNA was used as negative control (data not shown). In addition, unaltered expression of target genes upon treatment of each anti-miRNA in KCNQ1OT1-sgRNA-Huh7/SNU449 cells demonstrated that lncRNA-KCNQ1OT1 alters the target genes expression via miRNA. Thus, this data highlighted the functional network of lncRNA-miRNA-mRNA axis in HCC (Fig. 5J and S5I).

### Impact of KCNQ1OT1 on BMP signalling pathway

**Fig. 6:**
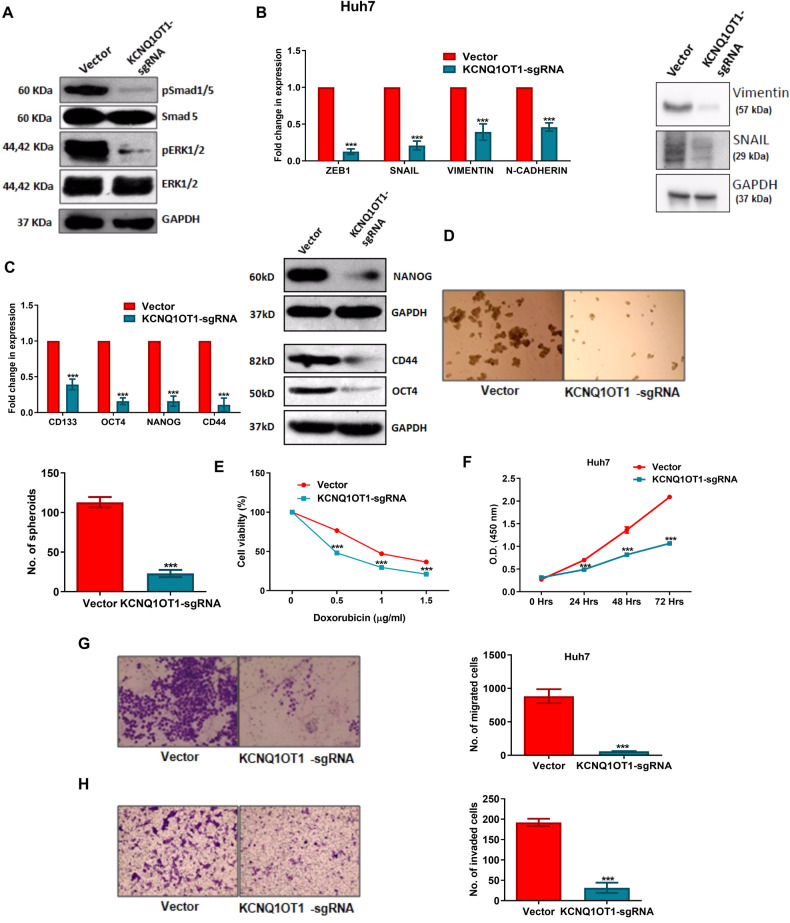
Characterization of KCNQ1OT1-sgRNA-Huh7 cells compared to Cas9-Huh7 cells. (**A**) Immunoblot analysis of the downstream BMP signalling genes SMAD5, p-SMAD5, ERK1/2, p-ERK1/2 was performed with the lysates of Cas9-Huh7 and KCNQ1OT1-sgRNA-Huh7 cells. qRT-PCR and Immunoblot analysis of (**B**) EMT markers and (**C**) stemness markers was done with Cas9-Huh7 and KCNQ1OT1-sgRNA-Huh7 cells. The effect of KCNQ1OT1 deletion on cancer progression was measured by quantifying (**D**) spheroid number, (**E**) chemosensitivity to doxorubicin, (**F**) proliferation, (**G**) migration, and (**H**) invasion properties of Cas9-Huh7 and KCNQ1OT1-sgRNA-Huh7 cells. *p* value was calculated using unpaired student’s t-test for **B**–**H**. *** indicates *p* value < 0.001.

**Fig. 7:**
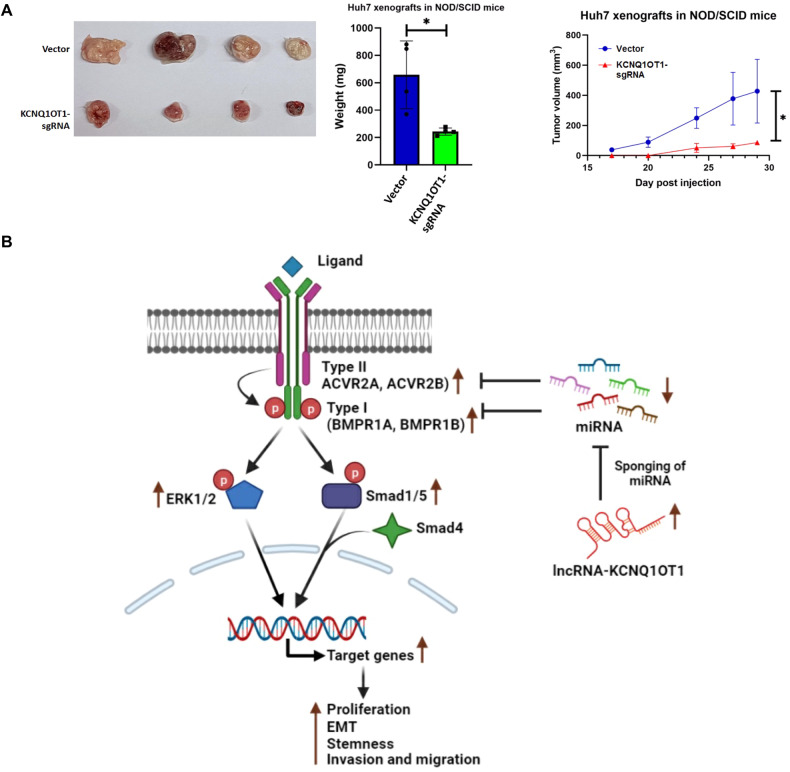
Mice Xenograft model with KCNQ1OT1-sgRNA-Huh7 cells. **A** NOD-SCID mice were injected with KCNQ1OT1-sgRNA Huh7 cells and Cas9-Huh7 cells into the right dorsal flank. At different time point, tumour volume and mass were determined. *p* value was calculated using Mann–Whitney test for **A**. * indicates *p* value < 0.05. **B** Graphical abstract of the study.

To understand the mechanism, immunoblot analysis was performed with Cas9-Huh7/SNU449 and KCNQ1OT1-sgRNA-Huh7/SNU449 cells and observed that the two major signalling molecules phospho-SMAD1/5 and phospho-ERK1/2 of BMP signalling pathway were drastically reduced in the absence of KCNQ1OT1 in KCNQ1OT1-sgRNA-Huh7/SNU449 cells compared to Cas9-Huh7/SNU449 (Figs. 6A and S6A). As a result, various phenotype of the cancer cells such as the expression of EMT and stemness markers, spheroid formation, chemoresistance, proliferation, migration and invasion etc. were significantly attenuated in KCNQ1OT1-sgRNA-Huh7/SNU449 cells compared to Cas9-Huh7/SNU449 cells (Figs. 6B–H and S6B–H). Furthermore, subcutaneous xenograft tumour model showed tumour growth arrest when KCNQ1OT1-sgRNA-Huh7 cells were injected to the right dorsal flank of NOD-SCID mice compared to the Cas9-Huh7 cells (Fig. 7A).

Collectively, our data suggest that KCNQ1OT1 is overexpressed in HCC and sequesters multiple beneficiary miRNAs inducing CSC-like features in cancer cells leading to chemoresistance (Fig. 7B). Hence, anti-KCNQ1OT1 treatment has robust potential to improve therapy in advanced HCC patients.

## Discussion

In this study, we uncovered the function of the five most downregulated tumour suppressor miRNAs, miR-424-3p, miR-136-3p, miR-139-5p, miR-223-3p, and miR-375-3p in HCC. Surprisingly, we observed that these miRNAs convergently inhibited signalling pathways regulating pluripotency of stem cells where targets were type-I and type-II BMP receptors (BMPR1A/BMPR1B and ACVR2A/ACVR2B) which function after tetramerization in the presence of BMPs [15, 17]. Restoration of each miRNA independently in HCC cell lines (Huh7 and SNU449) arrested CSC-like phenotypes and made cells sensitive to doxorubicin. Here, we have explored the BMPR1B and ACVR2A receptors for the first time in HCC and observed suppression of these two receptors independently impeded BMP signalling pathway and both Huh7 and SNU449 cells lost CSC-like features. As a result, HCC cells exhibited sensitivity to doxorubicin. Furthermore, using the advantages of bioinformatics tool, a 90Kb long lncRNA-KCNQ1OT1 was identified as a common regulator of all these miRNAs, and by performing series of in silico*,* ex vivo and in vitro experiments, it has been established that KCNQ1OT1-sgRNA-Huh7/SNU449 cells displayed slow proliferation, reduced stemness, & metastatic potential and increased sensitivity to chemotherapeutic drug. Additionally, there was visible shrinkage of tumour size in NOD/SCID mice implanted with these cells depicting strong potential of KCNQ1OT1 as a therapeutic target in HCC patients with poor prognosis.

Our transcriptome data of liver tissue samples from HCV-HCC patients resonate well with three GEO datasets of HCC (GSE21362, GSE40744, and GSE74618) which reported that miR-223-3p, miR-375-3p, miR-424-5p, miR-136-3p, and miR-139-5p were among the downregulated miRNAs in both HCV-HCC and HBV-HCC. BMP signalling pathway through BMPR1A/BMPR1B and ACVR2A/ACVR2B were commonly inhibited by each of these miRNAs. There is paucity of data related to this signalling pathway. Only a few studies described overexpression of BMP4 protein in HCC liver tissues compared to adjacent non-tumour tissue [18]. Wang X et al. reported that the expression of both BMPR1A/BMPR1B and BMPR2 mRNA are highly variable [19]. Though, we have not verified the level of BMP4, the receptors were mostly overexpressed in our HCC cohort and each miRNA has high potential to block these receptors and arrest phosphorylation of the two downstream signalling molecules SMAD1/5 and ERK1/2. This was also verified in Huh7 and SNU449 cells after treatment with BMPR1B-AS and ACVR2A-AS oligo. By recruiting cAMP-responsive element binding protein (CREB) and p300, phospho-SMAD1/5 has been shown to induce differentiation of CSCs while phospho-ERK1/2 triggers both proliferation and differentiation [20, 21]. Here, we observed that the suppression of BMPR1B/ACVR2A reduced EMT, stemness, chemoresistance and metastasis in HCC cells though it is contradictory to the observation of Zhou et al. [22] in colon cancer progression and metastasis [22]. Further study is required to understand this signalling in HCC.

A few studies have documented the function of lncRNA as competitive RNA for beneficiary miRNAs such as lncRNA-KCNQ1OT1 sponges miR-148-3p, and miR-149 to induce oncogenic axis via IGF1R, S1PR1, respectively in HCC [23, 24]. Therapeutic potential of anti-sense lncRNA has been also established in many studies e.g., lncRNA ITGB8-AS1 markedly reduced proliferation and tumour growth in colon cancer [25]. In consistence with this, we have identified and validated that the lncRNA-KCNQ1OT1 acted as competitive RNA for all the five miRNAs and deletion of KCNQ1OT1 in Huh7 and SNU449 cells completely attenuated CSC-like phenotype in cancer cells by arresting BMP signalling pathway which is one of the major stem cells signalling pathways [15]. There might be a possibility that this 90 kb long lncRNA has potential to sequester many more tumour suppressor miRNAs in HCC. In addition, reduced tumour size in NOD/SCID mice injected with the KCNQ1OT1-sgRNA Huh7 cells corroborated well with the above observations and depicting strong therapeutic potential of anti-KCNQ1OT1 along with other conventional therapy for clinical management of advanced HCC patients.

Therefore, this novel study suggests that as miRNAs are having pleiotropic functions, a complete understanding of the regulatory mechanisms of deregulated miRNAs with analogous functions may be essential for the successful application of ncRNA-based therapy. Since lncRNAs are having power to regulate multiple miRNAs simultaneously, it might be a better therapeutic target to restore the cumulative effect of multiple beneficiary tumour suppressor miRNAs and to prevent cancer.

## Materials and methods

(Detailed methods are available in the Supplementary file)

### Ethical clearance

The Human Ethics Committee of the Institute of Post Graduate Medical Education & Research (IPGME&R), Kolkata and the Animal Ethics Committee of National Centre for Cell Science, Pune had approved the study (Approval ID: Inst/IEC/2015/108 and IAEC/2022/B-414 respectively). Written informed consent was obtained from all the participants or legal guardians.

### Study subjects and samples included in the study

Fifty-nine treatment naive chronic hepatitis patients’ mono-infected with either HCV or HBV attending the hepatology clinic of School of Digestive and Liver Diseases, IPGME&R, Kolkata and Indraprastha Apollo Hospital, New Delhi were included in the study and categorized as Chronic Hepatitis B or C (CHB or CHC) (*n* = 21), Liver Cirrhosis (LC) (*n* = 17), and HCC (*n* = 21). Patients co-infected with HEV/HAV/HIV, having co-morbidities like chronic alcoholism, diabetes mellitus or unwilling to enrol in the study were excluded. Normal liver biopsy tissue was obtained from Gall bladder carcinoma patients (*n* = 11) during cholecystectomy from IPGME&R as routine evaluation of liver metastasis and confirmed after assessment of histology. Both blood and liver tissues were collected for proper disease evaluation and further study. Details of the study subjects, biochemical, and clinical data are presented in Table S1.

### Transcriptome profiling

Our small RNA transcriptome profile of liver tissue samples from HCV-HCC vs. normal individuals using Illumina platform has been deposited in the public domain as GSE140845 [26]. After analysis only downregulated miRNAs (log_2_fold change ≤−1.5, *P*_adj_ ≤ 0.05) were considered for this study. Public databases GSE21362, GSE40744, and GSE74618 were used for validation.

### Total RNA isolation and cDNA preparation

Total RNA was isolated from 1 mm^3^ tissue using TRIzol reagent following manufacturer’s protocol (ThermoFisher, #15596026). About 0.5 and 2.5 µg of RNA was used for cDNA synthesis of miRNA and mRNA using miScript PCR Starter Kit (Qiagen, #218193) or miRCURY LNA miRNA PCR Starter Kit (Qiagen, #339320) and RevertAid reverse transcriptase (ThermoFisher, #EP0441) respectively.

### Quantitative Real Time PCR (qRT-PCR)

Both miRNA and mRNA were quantified using PowerUp^TM^SYBR^TM^ Green PCR master mix (ThermoFisher, #A25742) and gene specific primers using QuantStudio 7 Flex RT-PCR machine (ThermoFisher). miR-103a-3p and 18 s rRNA were used as internal controls for miRNA and mRNA respectively. Fold change in expression of genes was calculated using the formula 2^-**Δ**Ct^, where ΔCt = (Ct _Gene_ – Ct _Internal control_). For tissue samples, log_10_2^-ΔCt^ value was plotted.

### Bioinformatics analysis

Targets of the miRNAs were identified using TargetScan/miRDB/micro-T-CDS. miRNet (https://www.mirnet.ca/) tool was employed to visualize the overall network of miRNAs. Targets were validated in LIHC and subjected to pathway analysis using KEGG (https://www.genome.jp/kegg/pathway.html).

LncBase V3.0-DIANA tool (https://diana.e-ce.uth.gr/lncbasev3/home) was used to predict the common lncRNA that binds to multiple miRNAs having 7-mer binding capacity with score ≥0.95.

### Cell lines and plasmid information

Huh7/SNU449 cell lines were STR profiled and tested for *Mycoplasma* by PCR. Cells were maintained in Dulbecco’s Modified Eagle Medium (DMEM, HiMedia, #AL111) with 10% FBS (GIBCO, #10082139) in CO_2_ (5%) incubator at 37 °C. Lipofectamine 2000 (ThermoFisher, #11668019) and TransIT-X2 Dynamic Delivery System (Mirus, #MIR6000) were used for transfection using manufacturer’s instructions.

Replication competent plasmids of HCV genotype3a, pS52/JFH1 (pHCV) [27], and pSVNeo2/HBV Dimer (pHBV) [28] were gifted by Jens Bukh, Copenhagen University Hospital, Denmark and Prof. Chiaho Shih, University of Texas Medical Branch, Galveston, USA respectively.

The sequence of each Pre-miRNA and 3′UTR of target genes were retrieved from UCSC Genome browser, amplified by PCR and cloned in pRNAU6.1^Neo^ vector and psiCHECK^TM^-2 (Promega, #C8021) vector at BamH1/HindIII and Xho1/Not1 sites respectively. Each PCR-Primer was appended with the corresponding restriction enzyme sites. Site directed mutagenesis kit (Agilent, #200523) was used to incorporate mutation in 3′UTR sequences following manufacturer’s protocol. A non-specific Pre-miRNA sequence (miR-c12) cloned in pRNAU6.1 [29] and mutated at the seed sequence was considered as pScramble pre-miRNA.

pAgo2-FLAG (Addgene, #21538) and pSpCas9(BB)-2A-Puro (PX459) (Addgene, #62988) [referred as pCas9] were gifted by Edward Chan, and Feng Zhang, respectively [30, 31]. All primers and oligo are listed in Table S2.

### CRISPR knock out cell generation

The sgRNA sequence for KCNQ1OT1 was selected using Genetic Perturbation Platform of Broad Institute (https://portals.broadinstitute.org/gpp/public/analysis-tools/sgrna-design) and the primers with minimum off-targets were chosen. After annealing of the primer pair, it was cloned into BbsI (NEB, #R3539) digested PX459 vector. The positive clones were confirmed by Sanger sequencing, transfected in Huh7/SNU449 cells and selected with puromycin.

### 3′UTR-Luciferase assay

Huh7 cells (2.5 × 10^4^) were seeded on 24-well and transfected with 200 ng of wild-type-3′UTR-Luciferase construct alone and with various plasmids/oligo e.g., pScramble Pre-miRNA (300 ng), pPre-miRNA (300 ng), pPre-miRNA (300 ng) + Scramble anti-miRNA oligo (20 pmol), pPre-miRNA (300 ng) + anti-miRNA oligo (20 pmol). One well was transfected with mutated-3′UTR-luciferase construct (200 ng) + pPre-miRNA (300 ng). After 36–48 h Luciferase assay was performed using Dual-Luciferase Reporter assay kit (Promega, #E2920) in GloMax® 20/20 Luminometer (Promega). Luciferase activity was normalized to the respective control. miRVana^TM^ miRNA inhibitor, Negative Control #1 was used as scramble anti-miRNA oligo (ThermoFisher, #4464076). Each experiment was performed twice in triplicate. The binding sequences of miRNAs to the target genes along with the site of mutagenesis are presented in Table S3.

### Immunoblot analysis

Huh7 (2.5 × 10^5^) and SNU449 (0.5 × 10^5^) cells were seeded on a 6-well plate and transfected with plasmid (3 µg) and/or anti-miRNA oligo (100 pmol). After 48 h, cell extract was prepared using RIPA buffer and about 20 μg of protein was subjected to polyacrylamide gel electrophoresis, and immunoblotted with specific primary and HRP-tagged secondary antibodies listed in Table S4. Pierce enhanced chemiluminescence (ECL) kit (Pierce, #SKU 34580) was used to detect specific protein. Full and uncropped western blots were added in the ‘Original Data file’.

### Wound-healing assay

Huh7 (5 × 10^5^)/SNU449 (1 × 10^5^) cells were transfected with plasmid (300 ng) and/or anti-miRNA oligo (20 pmol) in a 24-well plate. After 24 h of transfection, a thin scratch was created at the bottom of the plate and cell migration was monitored using EVOS^TM^ XL Core imaging system (Thermo Fisher Scientific) at 0, 24, 48, and 72 h. The data was analyzed using Image J software.

### Cell proliferation assay

Huh7/SNU449 cells were transfected with the required plasmids in a 24-well plate as described in wound -healing assay. After 24 h, cells were trypsinized and Huh7 (1 × 10^3^) and SNU449 (0.25 × 10^3^) cells were seeded on 96-well plate in triplicate. Cell proliferation was measured by adding Tetrazolium salt, WST1 (Sigma-Aldrich, #2210) at 0, 24, 48, and 72 h, and 0, 6, 12, and 18 h for Huh7 and SNU449 cells respectively in a microplate reader at 450 nm.

### Spheroid formation assay

After 24 h of transfection in 24-well following the above-mentioned transfection protocol, Huh7 (1 × 10^4^) and SNU449 (2.5 × 10^3^) cells were transferred on ultralow attachment plates (BD, USA) and allowed to grow in DMEM-F12 media (HiMedia, #SKU-AL140S) supplemented with 2% B27 (ThermoFisher, #17504001) and 20 ng/ml of epidermal growth factor (Gibco, #PHG0315) for 6 days. The size and number of the spheroids were documented using EVOS^TM^ XL Core imaging system (Thermo Fisher Scientific).

### Migration and invasion assay

After 24 h of transfection in a 24-well keeping the same protocol, Huh7 (1 × 10^4^) and SNU449 (2.5 × 10^3^) cells were transferred to the upper well of the Boyden chamber. After 48 h, the number of cells moved to the lower side of the membrane were fixed and counted by staining with crystal violet. FBS (30%) in the lower chamber was used as chemo-attractant. The upper chamber was coated with Matrigel (Corning, #356231) for invasion assay.

### Chemosensitivity assay

After 24 h of transfection with required plasmids in 24-well, Huh7 (1 × 10^3^) and SNU449 (0.25 × 10^3^) cells were transferred on 96-well plate in triplicate and treated with increasing doses of doxorubicin (0.5–2.5 μg/ml) (Sigma-Aldrich, #D1515) for 36 h and the number of cells survived were quantified using WST1 reagent (Sigma-Aldrich, #2210) in microplate reader at 450 nm.

### Biotinylation of KCNQ1OT1 fragments

The predicted binding regions of each miRNA to the lncRNA-KCNQ1OT1 were given in Table S5. The 800–1000 base-pair regions spanning each of the predicted miRNA-binding sites on lncRNA-KCNQ1OT1 were PCR amplified, cloned in pGEM-T Easy vector (Promega, #A1360), and sequence verified. To synthesize RNA from the cloned region of lncRNA, 1 μg of plasmid was subjected to in vitro transcription using T7 RNA polymerase (MEGAscript™ T7 Transcription kit; ThermoFisher, #A1330) for the synthesis of sense RNA and Sp6 RNA polymerase for the synthesis of anti-sense RNA (anti-sense RNA was used as negative control, data not shown). Synthesized RNA was then purified by ethanol precipitation and quantified using nanodrop. Next, 10–15 pmol of RNA was subjected to biotinylation using the Pierce^TM^ RNA 3′ End desthiobiotinylation kit (ThermoFisher, #20163). Biotinylated RNA was incubated with 100 μl of cell lysates prepared from Huh7 or SNU449 cells stably transfected with pCas9 or pCas9-KCNQ1OT1-sgRNA, and the resulting ribo-complex was pulled down with streptavidin magnetic beads following manufacturer’s protocol (Pierce, #20164). RNA from the pull-down was extracted using TRIzol and quantified by qRT-PCR.

### RNA Immunoprecipitation (RIP) assay

The plasmid pAgo2-FLAG (3 μg) was transfected in pCas9/pCas9-KCNQ1OT1-sgRNA Huh7/SNU449 cells and harvested 48 h post transfection and equal amount of protein were used for immunoprecipitation with anti-FLAG (Sigma-Aldrich, #F3165) and anti-IgG (ThermoFisher, #31143) antibody separately for overnight at 4 °C. Following precipitation of the RNA-protein complex with protein A/G agarose beads (Sigma-Aldrich, #P9424), the RNA was isolated using TRIzol and quantified by qRT-PCR.

### Mice experiment

Mice experiments were performed with Cas9-Huh7 and KCNQ1OT1-sgRNA-Huh7 cells injected subcutaneously into the right dorsal flank of eight weeks old NOD/SCID mice (*n* = 4). The tumour volumes were measured after 4 weeks using the following formula: *π*/6[(*d*1*d2) 3/2], where *d*1 and *d*2 are two different diameters dimensions of a tumour.

### Statistical analysis

Statistical analysis was performed using the GraphPad prism version 8. All the data were presented as mean ± standard deviation of three independent replicates. *P* value was calculated using unpaired Student’s t-test (for the data with Gaussian distribution) and Mann–Whitney test (for the data with Skewed distribution). Bonferroni correction (two-way ANOVA) was done for the grouped data analysis. *P* value <0.05 was considered as statistically significant.

### Supplementary information

Supplementary material

Original data file

## Data Availability

The data presented in the manuscript and the materials used for this study may be available upon request to the corresponding author.
