# Supplementary material for: Sponging of five tumour suppressor miRNAs by lncRNA-KCNQ1OT1 activates BMPR1A/BMPR1B-ACVR2A/ACVR2B signalling and promotes chemoresistance in hepatocellular carcinoma
Source: Cell Death Discov. 2024 Jun 8;10:274. doi: 10.1038/s41420-024-02016-0 (PMC11162467; doi:10.1038/s41420-024-02016-0)
Supplement: Supplementary file 1 — Supplementary material [file 41420_2024_2016_MOESM1_ESM.docx]

**Sponging of five tumour suppressor miRNAs by lncRNA-KCNQ1OT1 activates BMPR1A/BMPR1B-ACVR2A/ACVR2B signalling and promotes chemoresistance in hepatocellular carcinoma**

Swagata Majumdar *et al.*

Supplementary Tables and Figures

**Table of Contents**

Materials and methods Page 2-8

Table S1 Page 8-9

Table S2 Page 9-10

Table S3 Page 11

Table S4 Page 12

Table S5 Page 12

Table S6 Page 13

Table S7 Page 13-15

Fig S1 Page 15

Fig S2 Page 16

Fig S3 Page 17

Fig S4 Page 18

Fig S5 Page 19-20

Fig S6 Page 20

Uncropped Raw Western Data Page 21-33

**Materials and methods:**

**Study subjects**

Treatment naïve chronic hepatitis patients’ mono-infected with either HCV or HBV attending the hepatology clinic of School of Digestive and Liver Diseases, IPGME&R, Kolkata, India and Indraprastha Apollo Hospital, New Delhi, India were included in the study. Patients co-infected with HAV/HDV/HEV/HIV, having co-morbidities like chronic alcoholism, diabetes mellitus, autoimmune disorder etc. and unwilling to enrol in the study were excluded. Patients were categorized as Chronic Hepatitis B or C (CHB or CHC), Liver Cirrhosis (LC) and Hepatocellular carcinoma (HCC) after detailed assessment of clinical, biochemical, virological and histological evidences. Both CHC and CHB patients (n=21, n_HCV/CHC_=7, n_HBV/CHB_=14) were having high viral load (>10^4^ copies/ml), ALT>40 IU/L and with evidence of active necro-inflammation. Seventeen patients diagnosed with esophageal or gastric varices, portal hypertension, splenomegaly, ascites etc. were included as decompensated LC (n=17, n_HCV/LC_=7, n_HBV/LC_=10). HCC patients (n=21, n_HCV/HCC_=12, n_HBV/HCC_=9) were confirmed with triphasic CT scan and/or AFP value (>250ng/ml).

Normal liver biopsy tissue was obtained from Gall bladder carcinoma patients (n=11) during cholecystectomy at Gastro-Intestinal Surgery Department, IPGME&R as routine evaluation of liver metastasis and confirmed after assessment of histological, clinical and biochemical records. Detailed parameters were listed in **Table S1**.

**Sample collection**

Liver tissues were collected right after percutaneous biopsy or removal of diseased liver during transplantation in RNA later (Merck, #R0901) and in 10% formalin. RNA later tubes were kept for 24h at 4°C for proper penetration of the solution in the tissue and then preserved at -80°C. Formalin tubes were stored at room temperature for block preparation within a month.

**Bioinformatics analysis**

1. **miRNA target prediction and pathway analysis**

Predicted targets of the five miRNAs were retrieved using 3 Bioinformatics tools: TargetScan (<https://www.targetscan.org/vert_80/>) miRDB (<http://mirdb.org>), and micro-T-CDS (<https://dianalab.e-ce.uth.gr/html/dianauniverse/index.php?r=microT_CDS>). Targets found common in at least two of the mentioned bioinformatic tools with context score ≥0.25 were considered for further validation using viral TCGA-LIHC datasets and the gene list is presented in **Table S7**. Putatively overexpressed predicted targets, verified from TCGA-LISC datasets, of each miRNA were subjected to the pathway analysis by KEGG (<https://www.genome.jp/kegg/pathway.html>) and DAVID (<https://david.ncifcrf.gov/>) to identify the targeted pathways. The ‘Signalling pathways regulating pluripotency of stem cells’ was found to be commonly targeted by these five miRNAs. miRNet (<https://www.mirnet.ca/>) tool was employed to visualize the complete network of these five miRNAs and targets.

1. **Prediction of long non-coding RNA (lncRNA) binding to multiple miRNAs**

LncBase V3.0-DIANA tool (<https://diana.e-ce.uth.gr/lncbasev3/home>) was used to predict the lncRNA that could bind to multiple miRNAs. LncRNAs which were showing highest binding capacity to the five miRNAs with score ≥0.95 were considered and thus, KCNQ1OT1 was selected for validation. Detailed binding sequences were provided in **Table S5**. miR-375-3p was unresolved as the predicted binding site to KCNQ1OT1 was not available in the bioinformatic tool.

**Plasmid information**

1. Replication competent plasmid for HCV genotype 3a, pS52/JFH1 was gifted by Jens Bukh, Copenhagen University Hospital, Denmark. The JFH1 is a full-length replication competent HCV, genotype 2a clone. S52/JFH1 is the recombinant HCV clone of genotype 2a and 3a having core, E1, E2, p7 and NS2 of genotype 3a reference strain S52. pSV2neo-HBV dimer was shared by Prof. Chiaho Shih, formerly University of Texas Medical Branch, Galveston, USA. It was a head to tail dimer of HBV which could produce full-length HBV in cell culture.
2. **Cloning of pre-miRNA and 3’UTR sequences**

The required sequences of pre-miRNA and 3’UTR of target genes were retrieved from UCSC genome browser, amplified with primers appended with restriction enzymes from genomic DNA. It was digested with BamH1/HindIII and Xho1/Not1, and ligated to pRNAU6.1^Neo^ vector and psiCHECK^TM^-2 (Promega, #C8021) vector digested with the same restriction enzyme respectively. It was transformed in competent DH5α cells and positive clones were selected by PCR and sequence confirmed. As a scramble pre-miRNA, a non-specific miRNA (hsa-miR-c12) cloned in pRNAU6.1^Neo^ vector and mutated in the seed sequence was used.

1. **Site directed mutagenesis (SDM)**

To incorporate mutations in the 3’UTR sequences, SDM kit (Agilent, #200523) was used and manufacturer’s protocol was followed. In brief, forward/reverse primers were designed from same sequence having required mutations in the middle of the sequence, allowed to anneal to the wild-type 3’UTR plasmid, and amplified 12-18 cycles. It was digested with Dpn1 enzyme to cleave the wild type template and mutant plasmid was selected after transformation and sequence confirmation.

1. **Cloning of long non-coding RNA**

The binding regions of the respective miRNAs to the lncRNA-KCNQ1OT1 were predicted using LncBase V3.0-DIANA (**Table S5**). The region panning about 800-1000 base pairs of the predicted binding sites were amplified and cloned into the pGEM-T Easy vector (Promega, #A1360) having both T7 and SP6 promoter for *in-vitro* transcription. The lncRNA clones were sequence confirmed.

***In-vitro* transcription and biotinylation assay**

The KCNQ1OT1 clones (for each miRNA binding regions) in pGEM®-T easy vector were linearised using EcoRI digestion and 1 µg of linearised plasmid was subjected to *in-vitro* transcription using T7/Sp6 RNA polymerase and incubated for 5-6 hr according to the manufacturer’s protocol **(**MEGAscript® Kit, #A1330**).** Sense strand was transcribed by T7 RNA polymerase from the T7 promoter was selected for further study. Transcript (antisense strand) generated using SP6 polymerase was used as negative control for the biotinylation assay (data not shown). After transcription, 1µl TURBO DNase was added and incubated at 37ºC for 15 mins to cleave any residual plasmids in the reaction mixture. The reaction was stopped with ammonium acetate, and RNA was extracted using phenol-chloroform followed by alcohol precipitation. Next, 10-15 pmol of RNA was used to label desthiobiotin at its 3’ end according to the manufacturer’s instruction (Pierce™ RNA 3' End Desthiobiotinylation Kit, #20163). Labelled RNA was purified using Chloroform-isoamyl alcohol. Glycogen was used to precipitate the labelled RNA.

Empty cas9 vector and KCNQ1OT1-sgRNA stable Huh7 and SNU449 cell lysates were prepared using IP Lysis Buffer (Pierce, #87787**).** Streptavidin magnetic beads were washed multiple times with 20mM Tris and incubated with the labelled RNA from the previous step using 1X RNA capture buffer for 20 mins with agitation at room temperature. The beads were then collected by placing the tube in the magnetic strand, washed in 1X RNA capture and the above-mentioned cell lysates (100 μl) were mixed with the beads. The mixture was incubated at 4ºC for 1 hr with agitation according to the manufacturer’s instruction (Pierce™ Magnetic RNA-Protein Pull-Down Kit, #20164)**.** The beads were then collected by placing the tube on magnetic strand, washed with 1X washing buffer and RNA was extracted from the beads using TRIzol.

**CRISPR knock out cell generation**

The sgRNA sequence for KCNQ1OT1 was selected using Genetic Perturbation Platform of Broad Institute (<https://portals.broadinstitute.org/gpp/public/analysis-tools/sgrna-design>) and the primers with minimum off-targets were chosen. The sgRNA sequence was selected 20 nucleotides upstream of the protospacer adjacent motif (PAM sequence). After phosphorylation and annealing of the primer pair (oligos in thermal cycler at 37°C for 30 mins and 95°C for 5 mins, then ramp down to 25°C at 5°C/min), the annealed oligo pair was ligated to Bbs1 (NEB, #R3539) digested PX459 vector. The positive clone was sequence confirmed and transfected in Huh7 and SNU449 cells seeded on 24-well plates, and selected with Puromycin (10mg/ml) to get KCNQ1OT1-sgRNA cells.

**Immuno-blot analysis**

Total protein was isolated using RIPA buffer, and quantified using Bradford reagent (Merck, #SKUB6916). About 10-20μg of protein was subjected to polyacrylamide gel electrophoresis after boiling at 90^0^C for 5 min with 2X Laemmli buffer. Gel was transferred to PVDF membrane (GE healthcare, # 10600023) and blocked with 5% non-fat milk (MP Biomedical, #902887) 2h at room temperature and membrane was probed with specific primary antibody overnight at 4^0^C with gentle agitation. GAPDH-HRP and anti-Histone H3 antibody were used as loading control for whole cell lysates/cytoplasmic fraction and nuclear fraction respectively. Anti-rabbit-HRP-conjugated secondary antibody was used as required. Detailed antibody list is given in **Table S4**. Enhanced chemiluminescence (ECL) kit (Pierce, #SKU 34580) was used to detect specific proteins in autoradiograph or ChemiDoc imaging system (Bio-rad).

To extract nuclear and cytoplasmic fraction, Huh7/SNU449 cells grown in 6-well plates were harvested using 150μl 1X RSB buffer (10mM Tris, pH 7.4, 10mM NaCl and 3mM MgCl_2_, 0.05% NP40, 0.5mM DTT and protease inhibitors) and centrifuged at 2000 rpm for 10 mins. The cytoplasmic supernatant fraction was collected. Nuclear pellet was washed three times with 1X RSB and suspended in 100ul of lysis buffer (20mM HEPES, pH 7.4, 0.42M NaCl, 1.5mM MgCl_2_, 0.2mM EDTA and 25% v/v glycerol, 0.5mM DTT and protease inhibitors), kept on ice for 15-20 mins and centrifuged at 14000 rpm for 5 mins and supernatant was collected.

**RNA Immuno-precipitation (RIP) assay**

The plasmid pAgo2-FLAG was transfected in empty cas9 vector and KCNQ1OT1-sgRNA-Huh7 and SNU449 stable cells, seeded on 6cm dishes. Cells were harvested in ice-cold freshly prepared lysis buffer 48h post transfection. Proteins were estimated and equal amount of protein was used for immuno-precipitation with Anti-FLAG (Sigma, #F3165) and Anti-IgG (ThermoFisher, #31143) antibody separately overnight at 4°C with gentle agitation. Next day, the RNA-protein complex was precipitated with 40ul of protein A/G Agarose beads (Sigma, #P9424) and the RNA was isolated using TRIzol. qRT-PCR was used to quantify RNA. Each *in-vitro* experiment was performed thrice in triplicate.

**Xenograft Mice Model**

One and half million control or KCNQ1OT1-sgRNA-Huh7 cells in 50 µL basal DMEM were mixed with 50 µL growth factor reduced, phenol red-free Matrigel® (Corning, 356231) and injected into the right dorsal flank of eight weeks old male NOD/SCID mice (n=4 mice per group). The animals were monitored for growth of palpable tumours at regular intervals. Once palpable tumours were observed, the dimensions were measured using digital Vernier Caliper. At the termination of the experiment, the animals were euthanized by CO_2_ asphyxiation. The tumours were excised and collected in DPBS. Tumours were weighed and images were acquired. The tumours were snap-frozen in liquid nitrogen as well as fixed in 10% neutral buffered formalin. The tumour volumes were calculated using the following formula: π/6[(d1*d2)3/2], where d1 and d2 are two different dimensions of a tumour. Tumour volume and weight graphs were plotted and the statistics were calculated using GraphPad Prism version 8.

**Table S1:** Demographical and clinical parameters of samples included in the study.

| **Parameters** | **Normal**  **(n=11)** | **Virus infected Patient** | | | |
| --- | --- | --- | --- | --- | --- |
|  |  | **CHC**  **(n=21)** | **LC**  **(n=17)** | **HCC**  **(n=20)** | **P**  **value** |
| **Epidemiology** |  |  |  |  |  |
| Gender(M/F) | 11/0 | 19/2 | 15/2 | 19/1 |  |
| Age, years (Mean±SD) | 39±9.28 | 33±5.72 | 44±7.34 | 55±5.19 | NS^$^ |
| Child Pugh Score (A/B/C) | - | - | 4/10/3 | 0/13/7 |  |
| **Laboratory**  **Results** |  |  |  |  |  |
| Alanine Transaminase (IU/L) Median(range) | 36  (13-91) | 43  (25.1-531) | 55  (17-590) | 101  (43-254) | 0.01^#^ |
| Aspartate Transaminase (IU/L) Median(range) | 41  (16-72) | 39  (22-381) | 81  (19-160) | 92  (20-335) | 0.005^#^ |
| Total bilirubin (mg/dl) Median(range) | 0.8  (0.3-2.4) | 0.95  (0.5-2.1) | 2.69  (0.67-42.7) | 1.5  (0.7-50.61) | NS^#^ |
| Albumin  (g/dl) (Mean±SD) | 3.1±0.28 | 3.8±0.79 | 3.11±0.5 | 3.17±0.5 | NS^$^ |
| INR  (Mean±SD) | 1.1±0.05 | 1.1 ±0.42 | 1.72±0.25 | 1.99±0.18 | NS^$^ |
| Alpha fetoprotein (ng/ml)  Median (range) | - | - | 25.63  (12-159) | 988  (9-121000) | NS^$^ |
| Anti-HCV Antibody | - | Positive  (7) | Positive  (7) | Positive  (11) |  |
| HBV DNA  log7 copies±SD | - | 5±2  (14) | 5±2  (10) | 5±1.5  (9) |  |

^$^One way ANOVA or ^#^Kruskal Wallis test was used in case of parametric and non-parametric

distribution respectively for the data between the normal and all the groups of HCV and HBV

infected patients. p≤0.05 was considered as significant.

**Table S2:** List of primers used in the study

| **Primers** | **Sequence (5’ to 3’ direction)** |
| --- | --- |
| **Cloning primers for pre-miRNA** | |
| hsa-miR-223 BamH1 F  hsa-miR-223 HindIII R | CGGATCCTCAGAGTCCCCTCCATGACC  GAAGCTTCTTGTGAGAACTTGGTGCTTGG |
| hsa-miR-375 BamH1 F  hsa-miR-375 HindIII R | AGGATCCGCAGATGCGTTCAG TAAGCTTGGCTGGTGCTGAGAGGCC |
| hsa-miR-424 BamH1 F  hsa-miR-424 HindIII R | CGGATCCACTTGGAGTGAAGTGGCCTAGTC  GAAGCTTCAGCCTAGCCAGGAATACTGCC |
| hsa-miR-136 BamH1 F  hsa-miR-136 HindIII R | CGGATCCAGATCAGTGGGCAGCTCTTCC  GAAGCTTCGCCATCACAACCTTACCAAT |
| hsa-miR-139 BamH1 F  hsa-miR-139 HindIII R | CGGATCCGACACCCTTGGGAGGCACTGG  GAAGCTTGTTACTCCAACAGGGCC |
| **3’UTR cloning primers** | |
| ACVR2A Xho1F  ACVR2A Not1R | CTCGAGTGCGCCATCTGTGC  GCGGCCGCTCTTCTGACAGACA |
| BMPR1A Xho1F  BMPR1A Not1R | CTCGAGGCTTCTATTGCCATGAAC  GCGGCCGCCTCTTAAGGATGGGAT |
| BMPR1B Xho1F  BMPR1B Not1R | GCTCGAGGGAGTCTGGCACAACTCCAT  AGCGGCCGCCTTCTCTCAGTGCCGTTCCA |
| BMPR1B Xho1F  BMPR1B Not1R | CTCGAGACAAACACCGCCTGTCTAGG  GCGGCCGCACAAGCCATCTGCACGATCA |
| **Anti-sense miRNA** | |
| Anti-miR-223-3p | T*GGGGTATTTGACAAACTGACA |
| Anti-miR-375-3p | T*CACGCGAGCCGAACGAACAAA |
| Anti-miR-424-5p | T*TCAAAACATGAATTGCTGCTG |
| Anti-miR-136-3p | A*GACTCATTTGAGACGATGATG |
| Anti-miR-139-5p | A*CTGGAGACACGTGCACTGTAGA |
| **3’UTR Mutagenesis primers** | |
| miR-424-5p  ACVR2A mF  ACVR2A mR | AACTGGA**CCAGGTA**AGCTAA  TTAGCTTACCTGGTCCAGTT |
| miR-223-3p  ACVR2A mF  ACVR2A mR | TTTGAA**AAGTCAGA**TCAGAT  ATCTGATCTGACTTTTCAAA |
| BMPR1A mF  BMPR1AmR | ATTCAT**AGTCTACA**AACCAG  CTGGTTTGTAGACTATGAAT |
| miR-136-3p  BMPR1B mF  BMPR1B mR | ATTATG**CATCAAAA**AGTACT  AGTACTTTTTGATGCATAAT |
| miR-375-3p  BMPR1B mF  BMPR1B mR | CAATATG**CAAGACA**TGAAGG  CCTTCATGTCTTGCATATTG |
| **Expression Primers** | |
| BMPR1A F  BMPR1A R | GAGTTGCTGCATTGCTGACC  TCCACGATCCCTCCTGTGAT |
| BMPR1B F  BMPR1B R | CTCAGAGCTCAGGAAGTGGATC  CATGCCTCATCAACACTGTCTG |
| ACVR2A F  ACVR2A R | CTGCTCTATTCCTTGGTGCCACT  TTCCAGACACAACCAAATCTTCC |
| ACVR2B F  ACVR2B R | GCTACAGTTCATTGCTGCCG  TGTGGGCAATAGACGGCTTG |
| CD44 F  CD44 R | GTGGCAATGGAGCTGTGGAGGACAG  GCATCTGTTAAGTGTCCCAGCTC |
| CD133 F  CD133 R | GTGCTTGGTGCATTCATC  GGCCATCCAAATCTGTCC |
| Oct4 F  Oct4 R | CTTCAGGAGATATGCAAAGC  GAACCACACTCGGACCACAT |
| Nanog F  Nanog R | ACCAGTCCCAAAGGCAAACA  TCTGCTGGAGGCTGAGGTAT |
| Vimentin F  Vimentin R | GACAATGCGTCTCTGGCACGTCTT  CATTCTTCTGCCTCCTGCAGGTTCT |
| ZEB1 F  ZEB1 R | GTGACGCAGTCTGGGTGTAA  TGAGTCCTGTTCTTGGTCGC |
| Snai1 F  Snai1 R | CCCTCAAGATGCACATCCGAA  GCCTGGCACTGGTACTTCTTGACA |
| CDH2 F  CDH2 R | CACTGCTCAGGACCCAGATCGAT  GCCGAGTGATGGTCCAATTTCTCT |
| KCNQ1OT1 F  KCNQ1OT1 R | CCATGACCCCAGTGGAATATGTGC  GGGATGTGAGGATCAGGTAGGAAG |
| 18s F  18s R | GTAACCCGTTGAACCCCATT  CCATCCAATCGGTAGTAGCG |
| U6 snRNA F  U6 snRNA R | CTCGCTTCGGCAGCACA  AACGCTTCACGAATTTGCGT |
| **Anti-sense Oligo** | |
| BMPR1B | AAGCTGCCAGTTTAGTTCTTCC |
| ACVR2A | AGACGGCAAACGCCAACTTTG |
| **KCNQ1OT1 sgRNA cloning** | |
| sgRNA-F  sgRNA-R | CACCGGTATAGGGTCCGCACATGGT  AAACACCATGTGCGGACCCTATACC |
| **Sequencing of sgRNA** | TTTATGGCGAGGCGGCGG |
| **Cloning of KCNQ1OT1 fragment** | |
| miR-223-3p F  miR-223-3p R | CAGCTGGTGTTGTGGTGCAC  GCTGAATCTGCCAGTGCCTTG |
| miR-424-5p F  miR-424-5p R | GCTCATCATCACTGGCCATCA  ATGTCAGGCAGTGTAGTGCCTC |
| miR-136-3p F  miR-136-3p R | TGGGGTCATGGTGTGCTGCC  TGCCCTGAGCATGCACACAC |
| miR-139-5p F  miR-139-5p R | AGCTCGACATGGCTCTGGCA  TTCTTCCCCCACACCTTGCCCA |

**
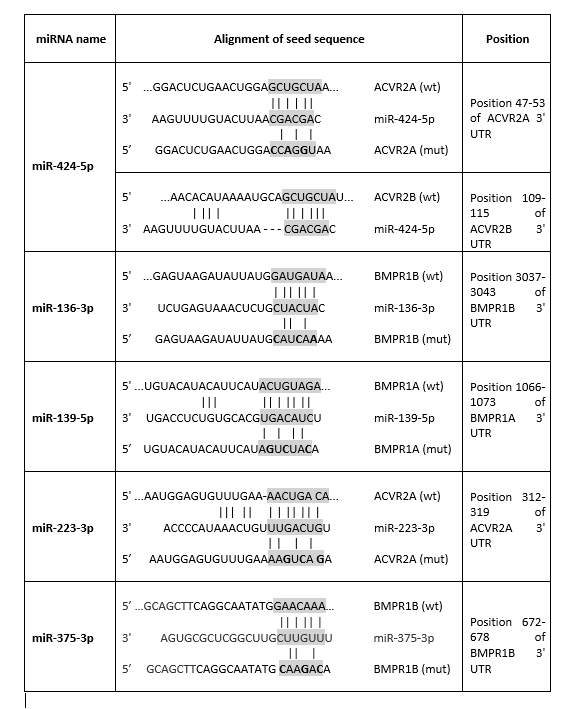
Table S3:** miRNA binding sites in the 3‘UTR regions of their target genes

**Table S4:** List of antibodies used in the study

| **Antibodies** | **Company** | **Origin** | **Clones** | **Catalogue No.** |
| --- | --- | --- | --- | --- |
| ACVR2A | ABclonal | Rabbit | Polyclonal | A1981 |
| BMPR1A | ABclonal | Rabbit | Polyclonal | A1816 |
| BMPR1B | ABclonal | Rabbit | Polyclonal | A2005 |
| CD44 | ABclonal | Rabbit | Polyclonal | A19020 |
| FLAG | Sigma | Mouse | Monoclonal | #F3165 |
| Histone 3 | BioBharti | Rabbit | Polyclonal | BB-AB0055 |
| Nanog | ABclonal | Rabbit | Polyclonal | A3232 |
| OCT4 | ABclonal | Rabbit | Polyclonal | A7920 |
| Smad4 | ABclonal | Rabbit | Polyclonal | A19116 |
| GAPDH-HRP | Cell Signaling | Rabbit | 14C10 | 3683 |
| ERK 1/2 | Cell Signaling | Rabbit | Polyclonal | 9102 |
| pERK1/2 | Cell signaling | Rabbit | Polyclonal | 9101 |
| Smad5 | Cell signaling | Rabbit | D4G2 | 12534 |
| pSmad1/5 | Cell signaling | Rabbit | 41D10 | 9516 |
| Goat anti-rabbit IgG-HRP | Santa Cruz | Rabbit | Polyclonal | sc-2004 |

**Table S5:** Predicted binding sequences of miRNA in KCNQ1OT1

| **miRNA name** | **Predicted sequence in KCNQ1OT1 (RNA) in 5’-3’** | **Predicted sequence in KCNQ1OT1 (DNA) in 5’-3’** |
| --- | --- | --- |
| hsa-miR-424-5p | UACCCAAAGGACUAUAAGUCAUGCUGCU | TACCCAAAGGACTATAAGTCATGCTGCT |
| hsa-miR-136-3p | UAUCAGUGGGGACUGUUCAGAAGAUGAUG | TATCAGTGGGGACTGTTCAGAAGATGATG |
| hsa-miR-139-5p | CUCUUGUACAGUGUUGGUGGGACUGUAAGA | CTCTTGTACAGTGTTGGTGGGACTGTAGA |
| hsa-miR-223-3p | UAGAAAGAUGAAAACUAUGA AAACUGACU | TAGAAAGATGAAAACTATGAAAACTGACT |

**Table S6:** Five downregulated miRNAs validated in public datasets

| Name | miR-424-5p | miR-136-3p | miR-139-5p | miR-223-3p | miR-375-3p |
| --- | --- | --- | --- | --- | --- |
| GSE21362  HCC=73  Normal=73 | log FC (-1.52)  p value=4.0e^-12^ | log FC (0.46)  p value=1.0e^-01^ | log FC (-1.42)  p value=4.3e^-10^ | log FC (-1.1)  p value=8.2e^-13^ | log FC (-2.3)  p value=1.3e^-14^ |
| GSE40744  HCC=9  Normal=12 | log FC (-2.19)  p value=1.6e^-05^ | log FC (9.78e^-02^)  p value=2.50e^-01^ | log FC (-2.4)  p value=8.3e^-05^ | log FC (-3.4e^-01^)  p value=5.70e^-01^ | log FC (-2.04)  p value=1.2e^-02^ |
| GSE74618  HCC=219  Normal=10 | log FC (-0.99)  p value=1.2e^-04^ | log FC (0.041)  p value=4.53e^-01^ | log FC (-3.36)  p value=1.8e^-13^ | log FC (-0.9)  p value=1.13e^-02^ | log FC (-2.01)  p value=5.8e^-05^ |

**Table S7:** Upregulated gene list validated in TCGA-LIHC dataset

| **Name of miRNA** | **Upregulated gene Validation in after TCGA-LIHC dataset** |
| --- | --- |
| **miR-424-5p** | SIX6, CCNE1, DNAJB4, TNFSF13B, LSM11, TMEM74B, PTH, CDCA4, MYB, BTLA, RBM6, OMG, TMEM100, EMC4, PURA, SIRT4, C1orf21, KCNJ2, SMAD7, TFCP2L1, KCNN4, WIPI2, PCDHA11, KIF23, USP25, PCDHAC2, MOB4, ARL3, SYS1, SLC35G1, RNF138, CAPZA2, BTAF1, CLDN12, HSPE1-MOB4, MYBL1, MEOX2, UBE4B, DYRK1B, GAREM2, RBMS1, POLR3F, MED26, SLC2A14, NDP, BTRC, ALOX12, SPAG7, WBP11,DCP1A, CPEB2, SLIT2, PCDHA3, ZNF449, CDK17, RFX3, CDC14A,KIF5A, FGF18, ZMYM2, HMGA1, CD28,PPM1D, ACVR2B, ACVR2A, SLC2A3P2, DENND6A, AREL1, ISLR, ATXN2, DYNC1I1, PAM, PARD6B, BZW1P1, FAM122A, USP42, EPHA7, BFAR, SOBP, QKI, EYA1, TLL1, BTF3, VAMP1, DMTF1, KIF5C, SESN1, RNF217, TSPYL2, FKBP1A, PLAGL1, FKBP1C, NAA15, ANLN, CLCN4, PHACTR4, IRAK2, ELMOD1, NISCH, CC2D1B, ZC3H11A, BICD1, NEBL,CUL2, OIP5, POU2F1, BTN1A1, RAF1, PCDHA5, PCDHA12, PPM1H, PRDM4, PCDHA9, PCDHA6,PCDHA1,PCDHA10, PCDHA8, PCDHA2, PCDHA13, PCDHA4, PCDHAC1,YTHDC1, LCOR, EFNB2, VAMP8, IGF1R,DNAJA2,LRIG2,ZC3H6,RREB1, LRP6, PNRC2, ITPRIPL1,ATP7A,NKD1,EED, FOXK1, CRK, PLCD1, TBP, EZH1, SIPA1L2, USP44, MTMR3, PDCD6IP, AKIRIN1, HNRNPA1, RGP1, LURAP1, SLC25A37, HIPK3, BPTF, THUMPD1, TTL, ZNF367, ADAMTS3, SALL3, GATA4, A4GNT, CDV3, SCN4B, VPS37C, FKRP, CX3CL1, TMC7,ERLIN2, STAG1, CNNM2,SETD1B, PPP1R11, LRRK1, SPOCD1, ZFHX4, PCSK5,SLC24A3, , RAB35, PRRC2C, MBD1, SEC24A, DYNC1LI2, DIAPH2, TRAK1,SEL1L3,GPRIN3, ZNF532, LTBP4, DEPTOR, KDR,NF1, TLE4, WNK3, EXOC5,FNDC3B, ADCY5, PCGF5, RNF14,RBM23,UBR3, DDX3X, PPP2R1B, PPIL4,SLC20A2,CALM1,DPH5, CDK1,VEZT, HCFC2, AKT3, APC, KRAS, RAF1,SKIL, SMAD7, WNT3A, WNT7A, ACVR2A, ACVR2B, BMPR1A, FGF2, FGFR1, FZD6, IGF1R, JARID2, OTX1, PIK3R1, ZFHX3, WNT4, WNT2B, AXIN2, |
| **miR-136-3p** | TGFB2, TRIAP1, RPGR, SCP2D1, COMMD2, MEPE, FABP2, VASN, RNF7, GPX7, UGT2A3, KCNJ3, ULBP1,C2CD2L, ZNF607, ANKEF1, GCA, CDC14A, FAM122B, TOR1AIP2, CENPP, AVL9, SSX2IP, SOX11, EMX2, SLC35A1, IGFBP5, HS3ST2, CARD17, DPP8, FAM216B, ZNF30, NCR2, ADAM7, FPGT, SPAG16, ZNF223, PELO, ZNF202, FBXL14, HOXB6, IQCE,IER3IP1, PKD2L2, SPANXN2, PKIG, NAA38, STK3, CALCB, ZNF285, RGS17, DDX18,PRKRIP1, RAB30, CCT3, USP13, PAFAH1B1P1, RNFT1, TPBG, GNPTG, RAET1L, ASCC3, SUMO3, KRTAP6-3,TFG, ZNF568, THAP5, HTR1D, SLC35A5, JRKL,EML1, CNIH1, ZBTB1, SMUG1, SNAP47, JMJD1C, PNPLA1, SPIN4, SKIV2L, TBL2, STC1, PDLIM3, STIM2, PAOX, CISD2, EAF2, SYCE1L, PHF21B,IRAK4, TAL2, TMED8, PRELID2, ZHX3, SGSH, SLC25A33, USP14, ECI2, MAPK9, LCORL, TTC23L, VCX3B, CADM1, FZD7, EMCN, PSMA4, CBLN2, EDIL3, ADO, TIMMDC1, S100A16, CGRRF1, CHST9, LIMA1, APOLD1, RPS6KA5, ATP6V1G1, DDHD2,CSNK1A1, GSTM3, SCG5, SUN3,FJX1, PLCXD1, FRRS1L, ZNF292, LYRM7, PLCB4, SHROOM2, RRN3, RRAGD, PARL, LRTM1, BUB3, CLCF1, RAB11FIP1P1, DEGS1, PHF20, NFIB, RCAN2, SLK, SNX1, HNRNPA3, PDE8B, SEC16B, ANXA5, RIMKLA, USP1, OCIAD2, BTNL3, IFNG, RGPD1, CDC40, PARP9, KCNJ1, CLVS1, PNPLA3, CPLX4, DUT, ZBTB8A, EIF5A2, MFSD8, BET1, MPZL2, CAMKMT, UBLCP1, ACTR3, BSND,TNFAIP8, GALK2, TXNRD3, CBWD1, TRIB2, CBWD6, CBWD3,BCAP29, SDHA, TUSC1, EP300, CBWD2, IGBP1, OARD1, COX5A, TMEM187, EPYC, RMI2,RALGAPB, SH3KBP1, DHX40, COX20, AKAP8, CRTAP, PALMD,CBX7, MTRNR2L1, HAS2, AMTN, ABHD3,SCPEP1,CHRNA1, OPRK1, CWF19L2, AFF3, PKP2,ZSCAN31, TAF1D, IDE, CANX, MRPL35,TCP11L2, PBK, TRAF1, HSPA12A,CPOX, POT1, GREM1,SNTB2, RAD1, MFSD1, RPAIN, KLF7, SLC35E4, DUSP12, NDUFAB1, CASP8, CTNND2, RRM1,TOR3A,TBCA,VTA1,EPHX1, LDHAL6A,CDK5RAP3,ANKRD44, MBOAT1, ELK3,SMG1,ZNF488, VIL1, TIAM2, MCFD2, HMGB1,ZNF451,RPS23, METTL21A, CCDC58, MRS2, PPIG, GLCE,MOB3B,ZFAND1,APC, SMAD5, WNT2B, BMPR1A, BMPR1B, FZD7, DVL3, GSK3B,PIK3R3. |
| **miR-139-5p** | ZBTB20, TBX1,ARL15, TGIF1, DPY30, TMF1, SCAPER, NR5A2, USP6NL, AKIRIN2, TPD52,MORN4, EBF1, PURA, PIEZO1, HOXB2, KBTBD2, MEIS2, TMED10, CSRNP2, PTAR1, TNPO1, NME7,EIF4G2,LMO4,ELOVL5, GDF10,QKI, LRFN5, AP1S2, MYCBP,GPR37, KIF13A, YAF2, CAMK2D,DUSP19,HDX, SRSF4,ZBTB34, LCOR, TCF4, GDE1, FAM76B, FMR1, PGM2L1, SSX2IP, UHMK1, PDE4D, NOL7, LRP12, PMP22, ARX, GDI2, HOXA9, TFEC, TOX, TMED7, TMEM132D, PPP2CA, PFDN6, DCBLD2, ATL2,CDK6,LANCL3, MRPS25, ATP2B2, PHF6, PSPH, SYT14, MAPK8, CUL3, HNRNPU, PDE3A, PDE4A, ZEB1, DYNLL2, MGAT4A, EDC3, RASGRF1, PTPRU,LRCH2,MBNL1,EMP2, NUFIP2,YWHAG, SCHIP1, TSPAN3, BMPR1A, MNT, PRDM16, HNRNPF, TIA1, KIF3A, GJA9, CDS2, REV1, ERN1, AZIN1, PITPNA, IQCJ-SCHIP1, IDE,FAM110B, LARP4B, TXLNG, BAZ2B, ZC3H12A, PRKD3, YWHAQ, KLHL23, PBXIP1, FBN2, KPNA4, SPCS3,BMPR1A, CTNNB1, DVL1,FZD3, IGF1R, KAT6A, ROCK1, SMAD2, HDAC2, PPP2CA, TGIF1. |
| **miR-223-3p** | CALML4, FBXW7, GTPBP8, ENPP5, SP3, APC, ABI2, TBC1D17, WDR62, PURB, LACC1, GTSF1, GALNT18, SNX24, CCDC149, HSP90B1, LRRC40, FAM199X, PDZD11, ATP7A, MBNL3, ARMCX1, ECT2, SLC37A3, LAYN, PAX6,GTDC1,CBX5, ARPP19, RRAS2,LMO2, RNF145,ACSL3,SPTLC2,DNAJB13, KPNA3, PKP4, SIAH1,RGS1,MEF2C,CTSV,ACVR2A,SYAP1,USP16,ARMC1,PHF19,PRDM1, SNCA, ARPC5L, PTBP2,SRP19, ZNF706, TBC1D15, SLC25A32, NDP,ATP10D, RWDD1, NUCKS1, WDR77, PEX16, TNNI3K, MID1IP1, FPGT-TNNI3K, ACTRT3, GPR22, RCN2, CDK17,NAA50,LYPD6,SREK1, KAT6A, UBE2A,KLF7,RASA1,VAMP2,SLC39A1,PROKR2, BRMS1L, SCAF8, CSPG5, TMEM178B,NFIB, RAP2A, FOXP1,SRPK2, CRIM1, SRSF10, ULK2, SDC2, SHOX2, ATG7, RNF34, RERG, XPR1, MMP16, PKNOX1, CBLB, ST3GAL1, CTNNA2, DESI2, SLC23A2, COPS8, POLR1C, SSRP1,GFPT1, MFSD6, POLR3E, TOP2B, ATP1B1, POMP, NXF1, ADCYAP1, PAX5,GPM6B, INPP4A, RAB8B, GLIPR1, ELK4, PSMA5, NUP210, CLSTN1, PURA, MTSS1, SPPL2A, FUBP3, TMED7, KCNMB4, NLRP3, SLCO1A2, NUTF2, ANKRD17, GABPB2, NUDT3, UHMK1, TMEM64, HHEX, POU2F1, ELP4, SCN1A, NRXN3, UBXN1,NDNF, USP42, PFN2,RSBN1L, STK39,MAP2K6, XKR6,SCARB1,SMARCD1, PHF20L1, FGFR2, FBXO28, DLEU1, TRMT2B, RGS9BP, RBMS2, MCMBP, ATP2B1, PLAGL2, SPRED1, USP6NL, TMEM170A, TSHZ3, SP1,RASSF4, VHL, RFTN2, TXLNG, AGO3, CCDC85C,APC, ACVR2A, FGFR2, FZD4, IGF1R, IL6ST, KAT6A, PAX6, FGF2,FGFR2,PIK3CA,RIF1, |
| **miR-375-3p** | ELAVL4,NDUFB9, EMC3, EIF4G3, RPN1,ZFP36L2, SHOX2, POU3F1,HNF1B, ATXN7, POU4F1, RASSF4, QKI, HOXA5, ISL2, YBX1, RBPJ,UBE2E2, MOB1A, LRP5, DESI1, IFT20, ZBTB20, PAX2, KCNE3, USPL1, ATP1B1, RBM24, CHSY1, MAL2, CAST, YAP1, LSM12, AP1S1, CCDC39, PAX6, SLC11A2, HSPH1, ELAVL2, PRKD1, ACSL3, CREBZF, MAP3K1, EIF4H, LYSMD3, JAK2,TIMM8A, MATR3, GMFB, ABI2, RNF126, ORMDL2, RASSF8, HOXA3, HLCS,PIAS1, CPPED1, HNRNPD, CADM1, CNIH4, OTP, UBE3A, KLF5,GRIK2,WWC2, PDGFA, LUC7L2, XPR1, TCF12, CCDC117,BC1D9, NLK, ANGEL2, RMDN3, PITPNA, UST, AP1AR, EIF1, ELK4, SYNJ2BP, RNASEH2B,JAK2, KLF4, BMPR1B**,** FZD4, PAX6. |

Green marked genes belong to ‘Signaling pathway regulating pluripotency of stem cells.

Though the expression of BMPR1B was more in HCC compared to normal but it was not significant in viral TCGA-LIHC transcriptome dataset. Further, in detailed data analysis revealed that its expression was highly variable among HCC samples. Transcriptome profile of our HCC samples also showed similar trend. It was overexpressed in 60% of our specimens (already written in the manuscript). Thus, we included BMPR1B in this study.

**
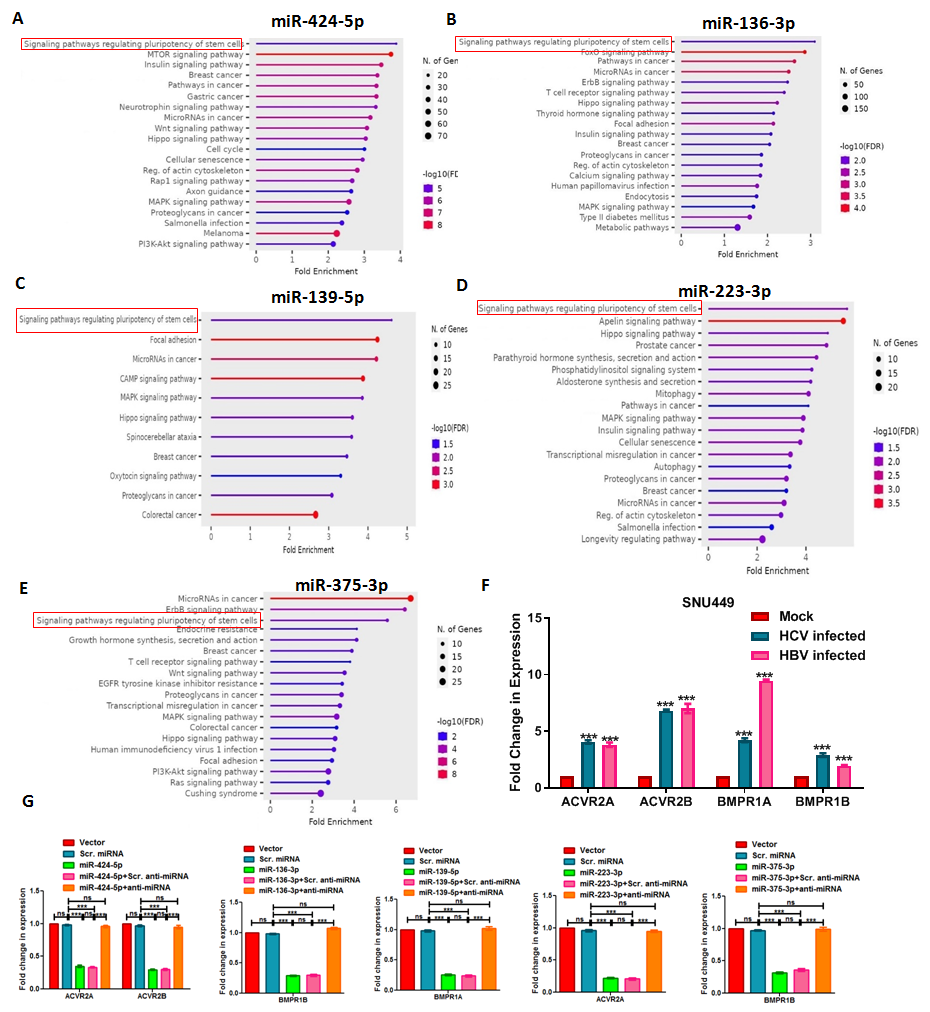
**

**Fig. S1**: **Pathway analysis of downregulated miRNAs. A-E** KEGG pathway analysis of individual targets of five miRNAs: miR-424-5p, miR-136-3p, miR-139-5p, miR-223-3p, and miR-375-3p respectively. **F** *In-vitro* qRT-PCR validation of the direct target genes of five miRNAs independently in HCV- and HBV- infected SNU449 cell line with respect to mock. **G** *In-vitro* qRT-PCR validation of individual target genes of five miRNAs in SNU449 cells transfected with pRNAU6.1 vector, pScramble pre-miRNA, pPre-miRNA, pPre-miRNA + Scramble anti-miRNA oligo, and pPre-miRNA + miRNA specific anti-miRNA oligo. p value was calculated using unpaired student’s t test for Fig. S1F, G. *** indicates p value < 0.001. ns means not significant.

**Fig. S2: BMP signalling cascade is regulated by five miRNAs individually. A, B** Immuno-blot analysis with lysates of SNU449 cells (A) transfected with pRNAU6.1 vector and five miRNAs independently, and (B) treated with scramble oligo, ACVR2A-AS, and BMPR1B-AS oligo were used assess the expression of total SMAD5, p-SMAD1/5, ERK1/2, and p-ERK1/2. GAPDH was used as internal loading control. **C, D** Huh7 and SNU449 cells were transfected with pRNAU6.1, pPre-miRNAs, and pPre-miRNA + miRNA specific anti-miRNA oligo independently and subjected to qRT-PCR for expression analysis of (C) EMT and (D) stemness markers. **E-F** Following same transfection protocol in SNU449 cells (E) spheroid numbers and (F) chemosensitivity to doxorubicin were measured. **G-I** SNU449 cells treated with either scramble oligo, ACVR2A-AS, and BMPR1B-AS oligo and assessed the expression of (G) EMT and stemness markers by qRT-PCR analysis and (H) spheroid number and (I) Chemosensitivity to doxorubicin. p value was calculated using unpaired student’s t test for Fig. S2C-F, and H-I, and two-way ANOVA for Fig. S2G. *, **, ***, **** indicate p<0.05, <0.01, <0.001, and <0.0001 respectively. ns indicates not significant.


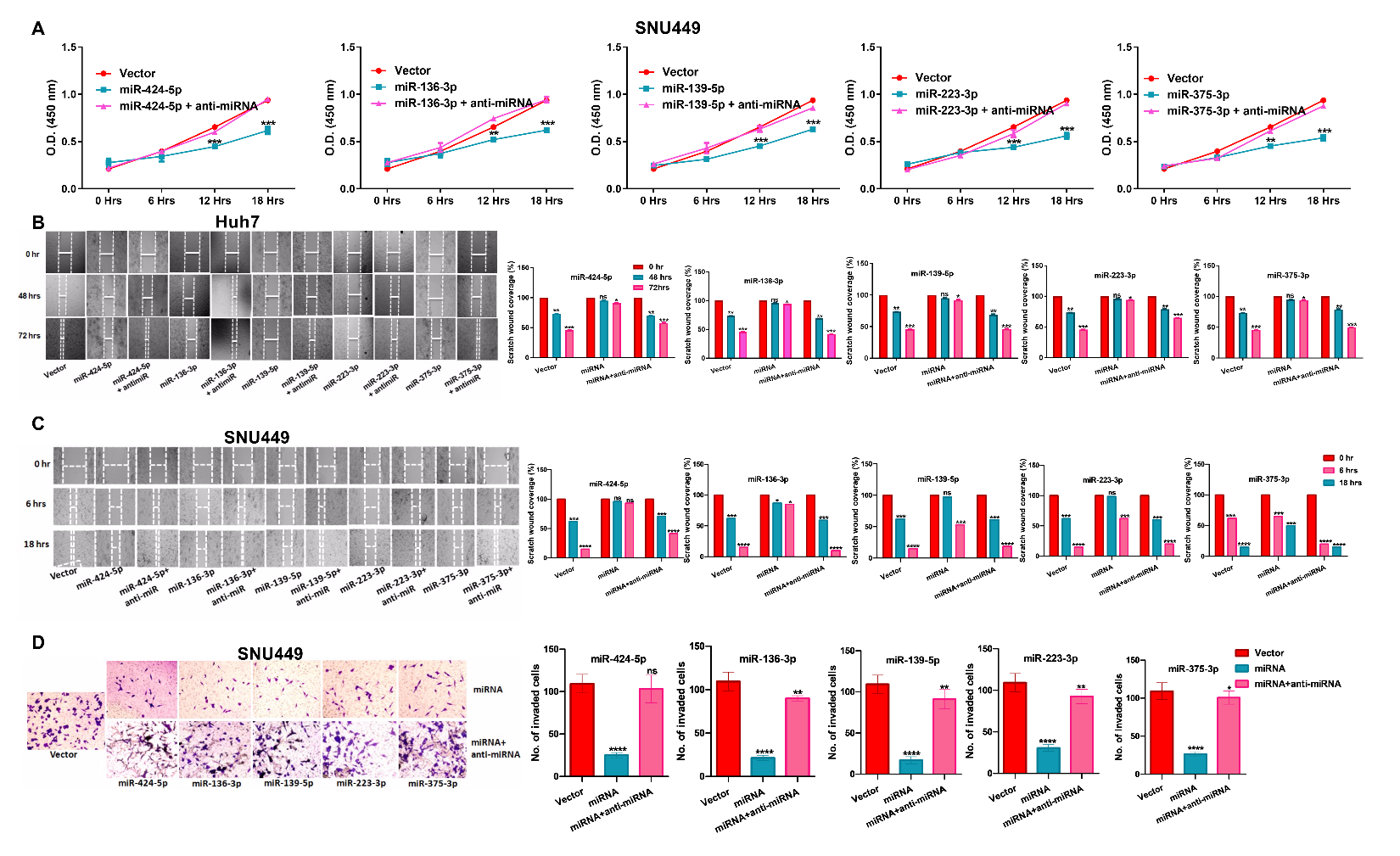


**Fig. S3: Role of five downregulated miRNAs in tumorigenesis.** **A-D** SNU449 and/or Huh7 cells were transfected with pRNAU6.1 vector, pPre-miRNAs, and pPre-miRNA + miRNA specific anti-miRNA oligo separately and assessed (A) cell proliferation in SNU449, wound healing or migration in (B) Huh7 and (C) SNU449 cells, and (D) invasion of SNU449 cells through Boyden chamber. p value was calculated using unpaired student’s t test for Fig. S3A-D. **, *** and **** indicate p<0.01, <0.001 and <0.0001 respectively. ns means not significant.

**
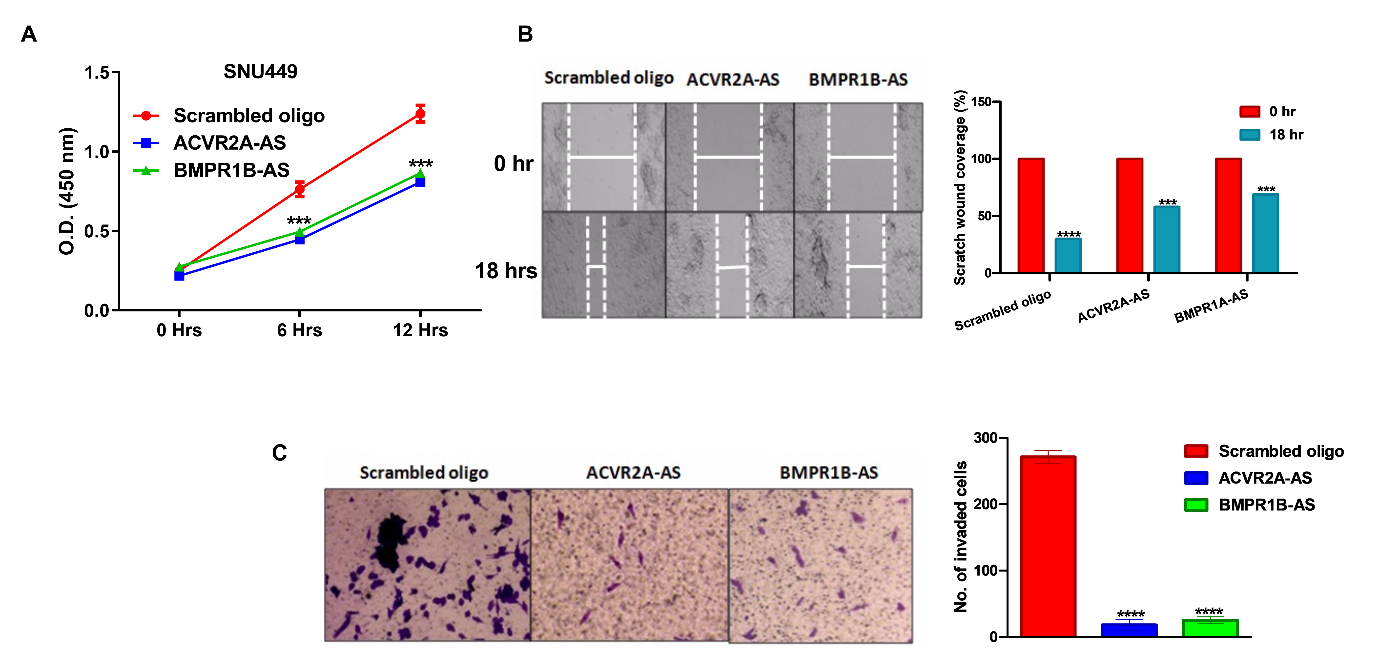
**

**Fig. S4: Role of BMP signaling receptors in tumorigenesis. A-C** SNU449 cells were treated with either scramble oligo, ACVR2A-AS, and BMPR1B-AS oligo and verified (A) cell proliferation, (B) wound healing or migration of cells, and (C) invasion of cells through Boyden chamber. p value was calculated using unpaired student’s t test for Fig. S4A-C. *** and *****, indicate p< 0.001 and <0.0001 respectively. ns means not significant.





**Fig. S5: LncRNA-KCNQ1OT1 is the regulator of five downregulated miRNAs. A-C** qRT-PCR validation of (A) five pre-miRNAs in control and HCC liver tissue specimens, (B) KCNQ1OT1 expression in both HCV- and HBV-infected SNU449 cells vs. vector cells, and (C) KCNQ1OT1 expression in nuclear and cytoplasmic fractions of the Huh7 and SNU449 cells. **D** Pearson-correlation for each of the five miRNAs and KCNQ1OT1 expression in HCC liver tissue specimens. **E, F** Cas9-SNU449 cells and KCNQ1OT1-sgRNA-SNU449 cells were used to verify the expression of (E) KCNQ1OT1 and five miRNAs by qRT-PCR*,* and (F) target genes, ACVR2A, ACVR2B, BMPR1A, and BMPR1B by qRT-PCR and immuno-blot analysis. **G** Cas9-SNU449 and KCNQ1OT1-sgRNA-SNU449 cells were transfected with pAgo2-FLAG and immune-precipitated with anti-FLAG/anti-IgG antibody, and enriched miRNAs in immuno-complex were quantified by qRT-PCR. **H** *In-vitro* KCNQ1OT1-miRNA interaction was studied by incubating biotinylated KCNQ1OT1 with either Cas9-SNU449 or KCNQ1OT1-sgRNA-SNU449 cell lysate. The complex was pulled down with streptavidin magnetic beads and enrichment of miRNA was verified by qRT-PCR. **I** qRT-PCR analysis of direct target genes of miRNAs by treating Cas9-SNU449 and KCNQ1OT1-sgRNA-SNU449 with miR-specific anti-miRNA oligo. p value was calculated using Mann-Whitney test for Fig. S5A, and unpaired student’s t test for Fig. S5B, and E-I. *** indicate p value < 0.001 and ns means not significant.





**Fig. S6: Characterization of KCNQ1OT1 Knockdown cells compared to control. A-H** Cas9-SNU449 and KCNQ1OT1-sgRNA-SNU449 cells were subjected to (A) immuno-blot analysis of SMAD5, ERK1/2, pSMAD1/5 and pERK1/2, qRT-PCR and immuno-blot analysis of (B) EMT and (C) stemness markers, (D) spheroid number, (E) chemosensitivity to doxorubicin, (F) cell proliferation, (G) wound healing, and (H) invasion assay using Boyden chamber. Wound healing was also done with KCNQ1OT1-sgRNA-Huh7 cells. p value was calculated using unpaired student’s t test for Fig. S6B-H. *, **, *** and ***** indicate p<0.05, <0.01, <0.001 and <0.0001 respectively and ns means not significant.

**Fig 6A**
