# Supplementary figures and images for: Sponging of five tumour suppressor miRNAs by lncRNA-KCNQ1OT1 activates BMPR1A/BMPR1B-ACVR2A/ACVR2B signalling and promotes chemoresistance in hepatocellular carcinoma
Source: Cell Death Discov. 2024 Jun 8;10:274. doi: 10.1038/s41420-024-02016-0 (PMC11162467; doi:10.1038/s41420-024-02016-0)

**Raw Data File**


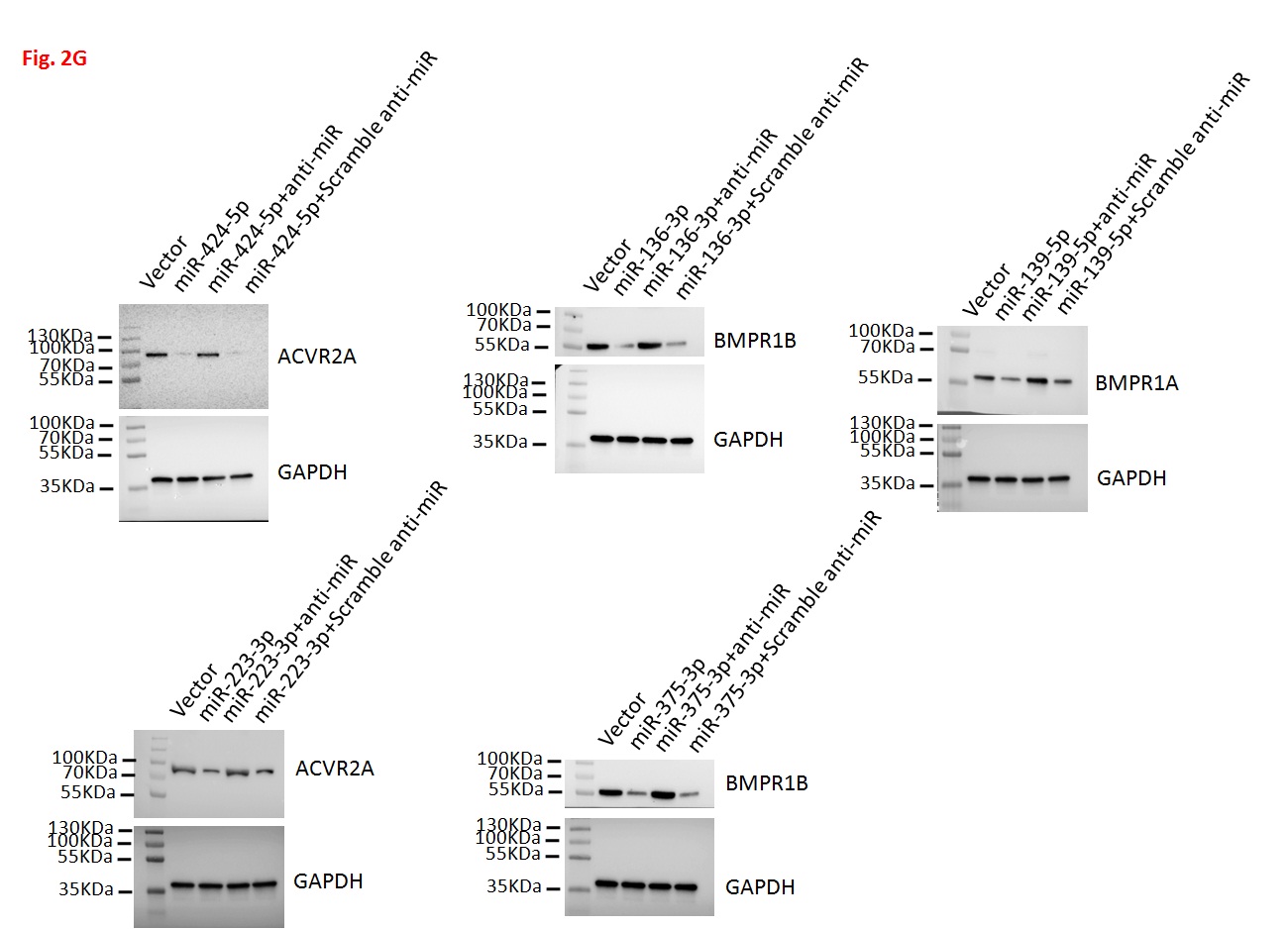


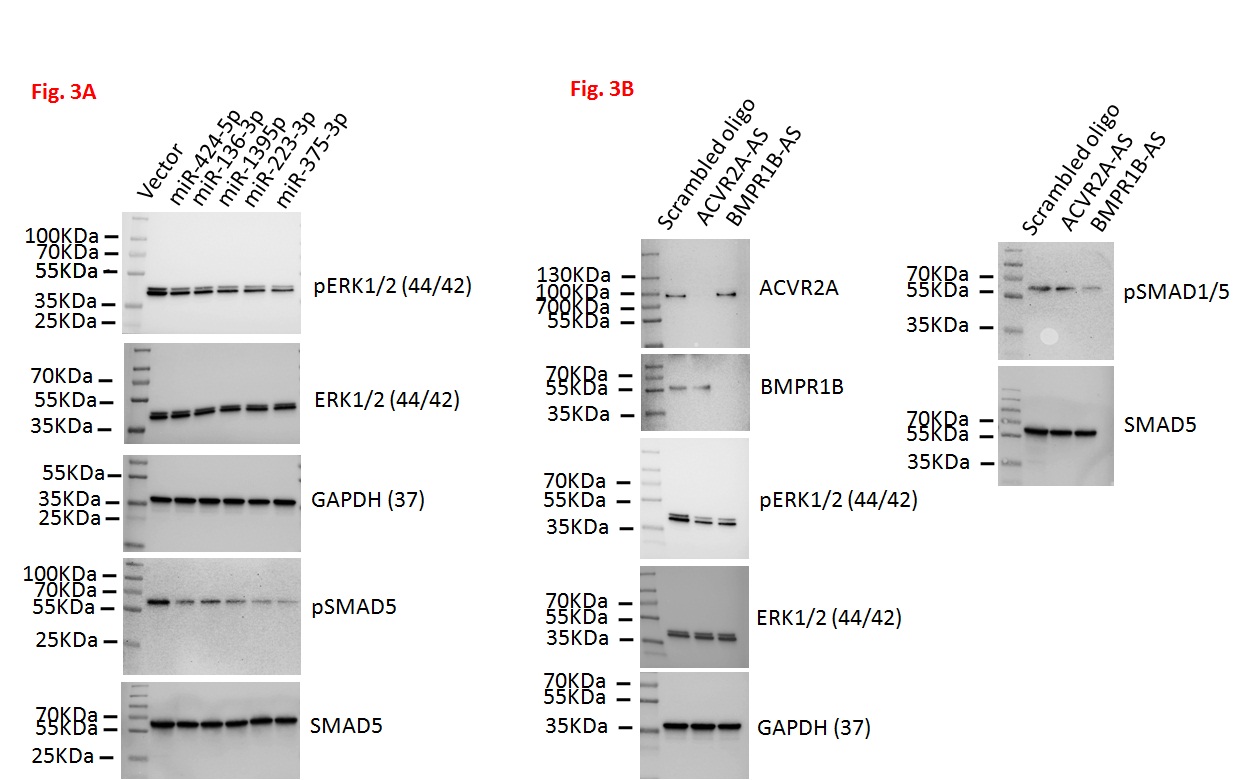


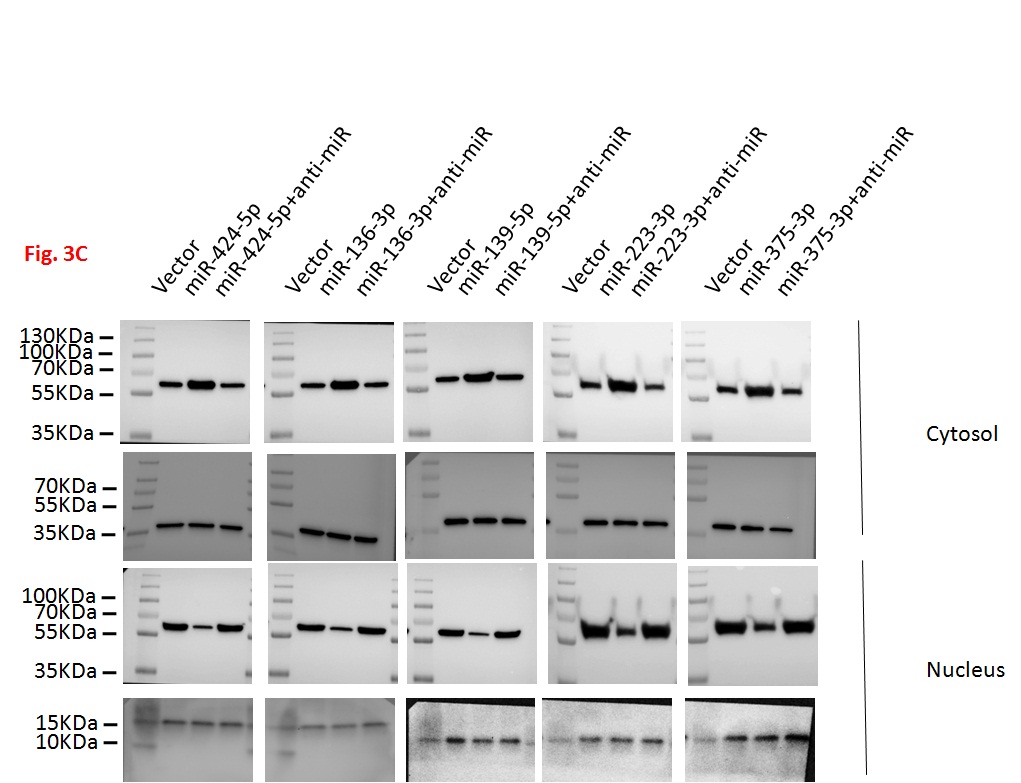


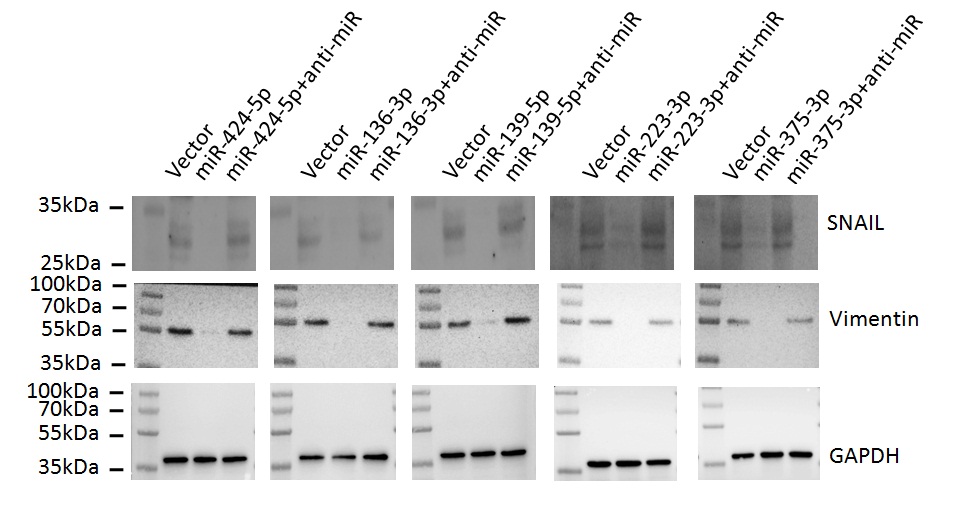


**Fig 3D**


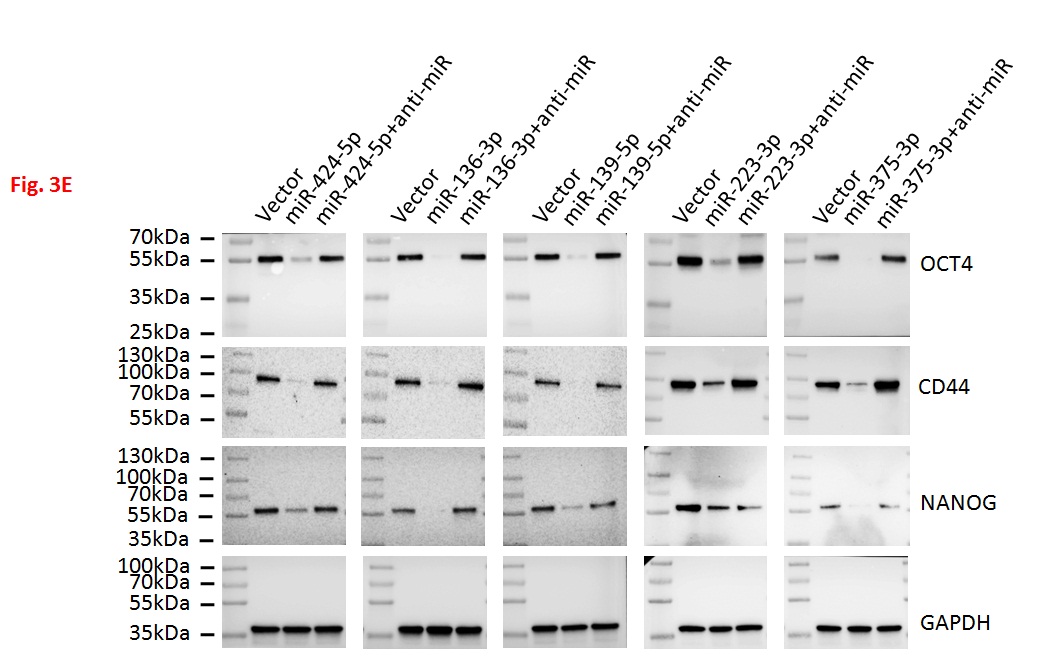


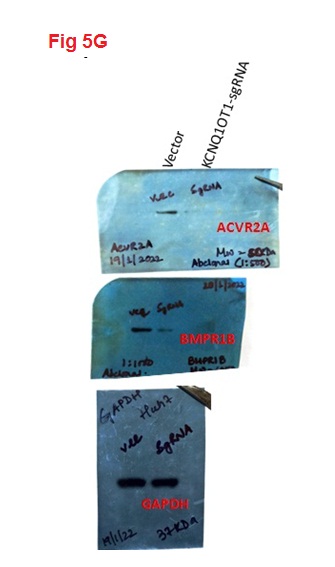


**Fig 6A**


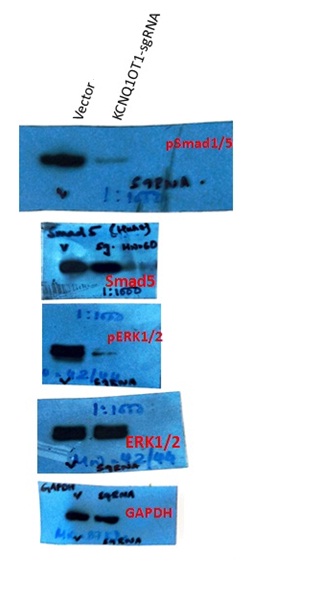


**Fig 6A**


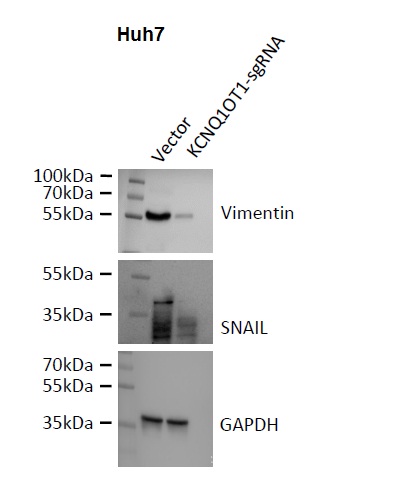


**Fig 6B**


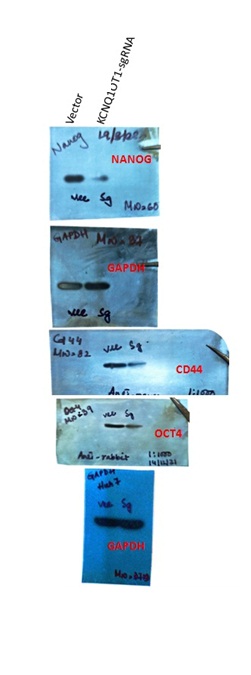


**Fig 6C**


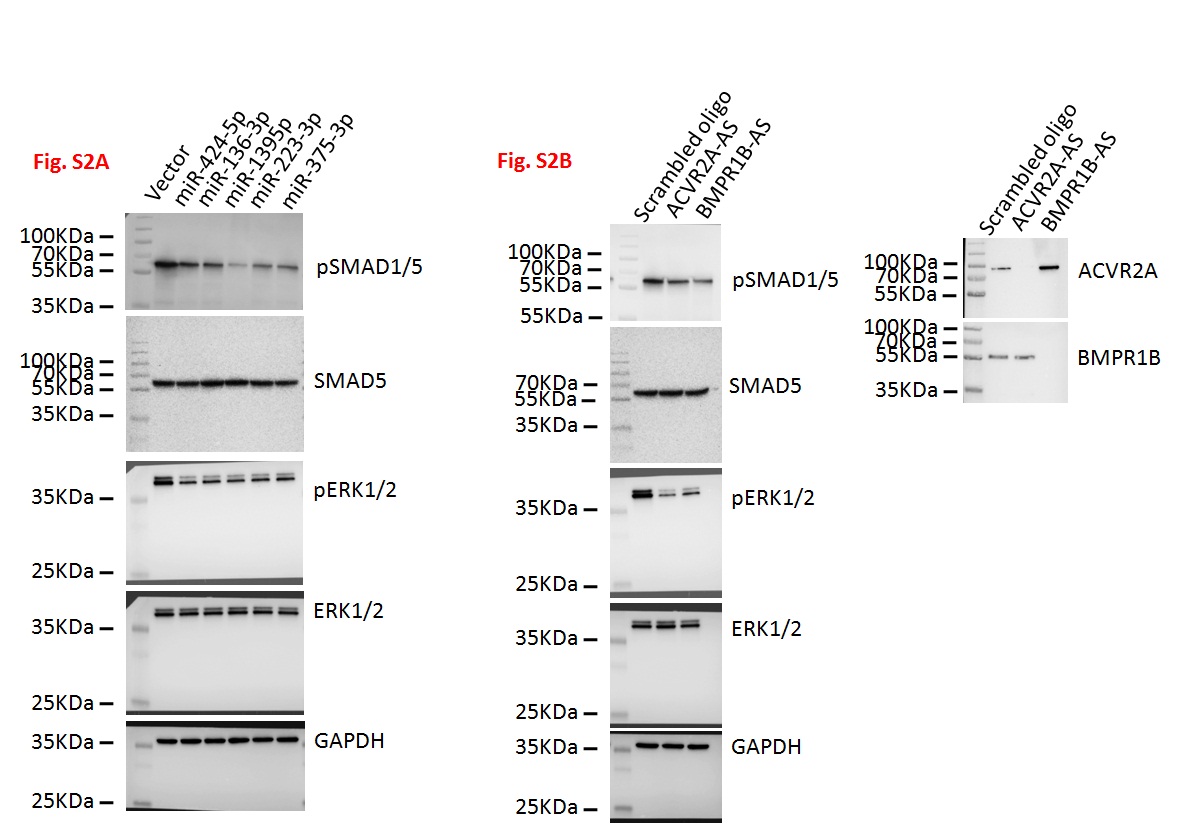


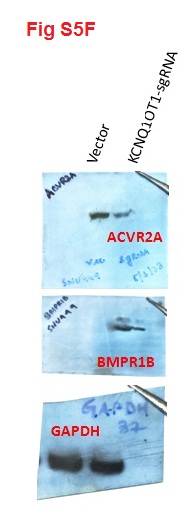


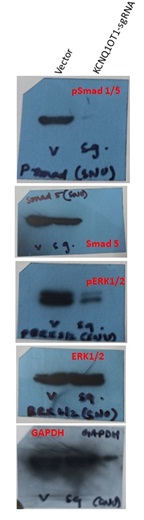


**Fig S6A**


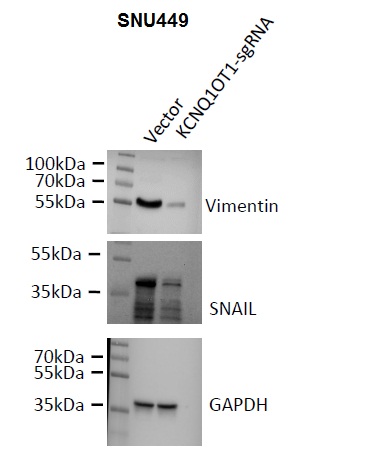


**Fig S6B**


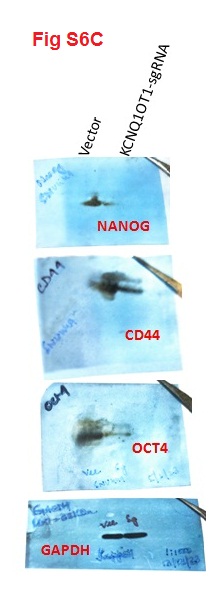

Supplement: Supplementary file 2 — Original data file [file 41420_2024_2016_MOESM2_ESM.docx]
